# PE-STOP: A versatile tool for installing nonsense substitutions amenable for precise reversion

**DOI:** 10.1016/j.jbc.2023.104942

**Published:** 2023-06-19

**Authors:** Ziguo Song, Guiquan Zhang, Shuhong Huang, Yao Liu, Guanglei Li, Xianhui Zhou, Jiayuan Sun, Pengfei Gao, Yulin Chen, Xingxu Huang, Jianghuai Liu, Xiaolong Wang

**Affiliations:** 1 International Joint Agriculture Research Center for Animal Bio-Breeding of Ministry of Agriculture and Rural Affairs, College of Animal Science and Technology, Northwest A&F University, Yangling, Shaanxi, China; 2 Zhejiang Lab, Hangzhou, Zhejiang, China; 3 State Key Laboratory of Pharmaceutical Biotechnology and MOE Key Laboratory of Model Animals for Disease Study, Model Animal Research Center at Medical School of Nanjing University, Nanjing, China; 4 School of Life Science and Technology, ShanghaiTech University, Shanghai, China; 5 Key Laboratory of Livestock Biology, Northwest A&F University, Yangling, Shaanxi, China; 6 CAS Center for Excellence in Molecular Cell Science, Shanghai Institute of Biochemistry and Cell Biology, Chinese Academy of Sciences, Shanghai, China

**Keywords:** prime editing, stop codon, nonsense mutation, off-target, base editing, PE-STOP, gene inactivation

## Abstract

The rapid advances in genome editing technologies have revolutionized the study of gene functions in cell or animal models. The recent generation of double-stranded DNA cleavage–independent base editors has been suitably adapted for interrogation of protein-coding genes on the basis of introducing premature stop codons or disabling the start codons. However, such versions of stop/start codon–oriented genetic tools still present limitations on their versatility, base-level precision, and target specificity. Here, we exploit a newly developed prime editor (PE) that differs from base editors by its adoption of a reverse transcriptase activity, which enables incorporation of various types of precise edits templated by a specialized prime editing guide RNA. Based on such a versatile platform, we established a prime editing–empowered method (PE-STOP) for installation of nonsense substitutions, providing a complementary approach to the present gene-targeting tools. PE-STOP is bioinformatically predicted to feature substantially expanded coverage in the genome space. In practice, PE-STOP introduces stop codons with good efficiencies in human embryonic kidney 293T and N2a cells (with medians of 29% [ten sites] and 25% [four sites] editing efficiencies, respectively), while exhibiting minimal off-target effects and high on-target precision. Furthermore, given the fact that PE installs prime editing guide RNA–templated mutations, we introduce a unique strategy for precise genetic rescue of PE-STOP-dependent nonsense mutation *via* the same PE platform. Altogether, the present work demonstrates a versatile and specific tool for gene inactivation and for functional interrogation of nonsense mutations.

The CRISPR–Cas9 genome editing technology, empowered by the original effector mechanisms in prokaryotic adaptive immunity, has revolutionized the biological researches since its emergence 10 years ago (1). One of the major applications of the technology is to establish gene knockouts, where the programmable Cas9 nuclease is harnessed to induce double-strand breaks (DSBs) and to drive subsequent frameshift, deletional, or donor DNA–directed mutations (2, 3). Despite the wide uses of such approaches, their reliant on the generation of DSBs poses challenges. The undesirable consequences of DNA damage–induced cellular toxicities (4, 5, 6), compromised genome integrity (7, 8, 9), and mixed editing products can complicate the investigation of gene functions in cell models and screening experiments. The more recently emerged base editors (BEs) represent a generation of DSB-independent genome editing tools. In principle, the cytosine and adenine BEs (CBE and ABE) are each constructed by the fusion of a cytidine or adenosine deaminase to a Cas9 nickase. CBE and ABE are poised to effectively trigger targeted C:G to T:A and A:T to G:C mutations, respectively, without the requirements of causing DSB or frameshifts (10, 11). Because of their abilities to install certain codon changes, the BEs have been adapted to program gene inactivation *via* introducing premature stop codons (*e.g.*, CBE-dependent CRISPR-STOP and iSTOP tools) or disruption of the start codon (*e.g.*, ABE-dependent i-Silence tool) (12, 13, 14, 15). Such strategies have been validated to enable the generation of gene-inactivated mouse models and the establishment of large-scale loss-of-function studies (16, 17, 18, 19). Nevertheless, the applicability of such stop/start codon–oriented knockout strategies is dependent on the sequence context of the targeted codons. A minor but visible fraction of the protein-coding genes may not be suited for the CBE/nonsense mutation–dependent gene inactivation, because of the unavailability of convertible sites within the early part of the coding sequences (CDSs) (12). On the other hand, the ABE-dependent disabling of start codons is directly under the restraints of whether the target bases are positioned within an ABE activity window (14). Furthermore, because of the adoption of deaminase activities, the aforementioned strategies can engage off-target (OT) editing at the genome and transcriptome levels (20, 21). It is also worth pointing out that for the important subset of nonsense alleles potentially with gain of functions (22), the current CBE-dependent strategies apparently lack the desirable coverage for their precise installation. Moreover, the prevalence of bystander editing besides the intended position by CBE (indeed characteristic of both BEs (23)) may further complicate the effort of studying such mutations.

Subsequent to the development of BEs, the prime editors (PEs) have been established as another version of highly versatile DSB-independent tools for precise genome editing. Empowered by the nCas9 (H840A)-anchored reverse transcriptase (RT) activity and a specialized mutation-encoding prime editing guide RNA (pegRNA), PE can precisely achieve all possible base transitions, transversions, and small-block genetic modifications within a flexible editing window (24). To potentiate the incorporation of reverse-transcribed edits, an additional component of nicking single-guide RNA (sgRNA) is supplemented to the basic PE (PE2) to constitute the PE3 platform. PEs have been harnessed to establish the whole-organism genetic models in various species including zebrafish (25), mice (26), and rice (27). Their applications have also been extended to studies of disease models and to saturation mutagenesis in genes of interest (28, 29, 30). Likely because of the requirement of multiple events of strand annealing through the prime editing process, PE features low OT activities (24, 31, 32). On the other hand, given the overall suboptimal efficiencies by PE3, intensive efforts have been made for development of enhancement strategies. In this regard, besides the designs to improve pegRNA stability (33, 34, 35, 36), modifications of PE architectures (*e.g.*, PEmax, PE2∗, and CMP–PE–V1) can serve to further enhance prime editing activities (29, 37, 38, 39).

In this study, we harness PE to develop a versatile gene targeting method termed PE-STOP. Owing to its substantially expanded genome coverages, reduced global OT rates and higher editing purities over the BE-dependent tools, the PE-STOP offers unprecedented opportunities for not only empowering gene inactivation but also precisely installing nonsense mutations with potentially *de novo* activities. The prospect of rescuing many PE-STOP-targeted mutations with a subsequent round of prime editing represents an additional advantage for this platform. Collectively, our work establishes PE-STOP as a promising tool for functional genomic studies.

## Results

### The design principle for the PE-STOP tool

**Figure 1:**
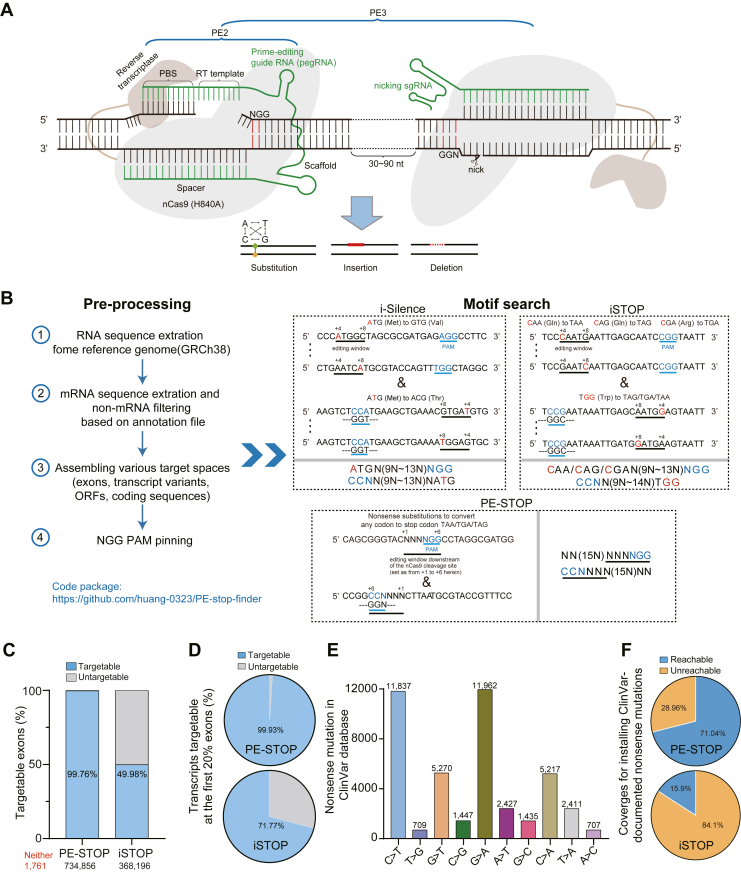
**The PE-STOP strategy is predicted to feature expanded genome coverages for gene inactivation and precise installation of nonsense mutations.***A*, a schematic view for the targeting actions by PE is shown. The nCas9 and reverse transcriptase motifs of PE are indicated. The nicked target DNA is depicted, with the PAM sequence (NGG) indicated. The pegRNA molecule is shown in *green*. The segments corresponding to the spacer, scaffold, RTT, and PBS are respectively marked. The *left portion* summarizes the PE2 components. For PE3, a nearby nicking sgRNA (canonical guide RNA) targeting the opposite strand is depicted on the *right*. PE enables installation of base conversions, small insertions, and small deletions. *B*, overall workflow for bioinformatic identification of PE-STOP, iSTOP, and i-Silence targetable sites in the human genome (under NGG PAM) is presented. The assembly of various target spaces is summarized on the *left*. Queries would be respectively made against all genes, transcripts, exons, and predicted ORFs. The sequence motifs used for searching editable sites by various editors are presented within the *box* on the *right*. In these boxes, examples of target sequences and generalized motifs are both presented. *C*, predicted percentages of human exons targetable by PE-STOP or iSTOP are presented. The targetable portions are shown in *light blue*, whereas the nontargetable portions are shown in *gray*. The numbers of exons targetable by the PE-STOP or iSTOP are marked under the *columns*. The number of exons untargetable by either strategy is denoted in *red*. *D*, the coverage of early exons (the number of exons included in each query = 1/5 × total number of exons in a given transcript, rounded up to the next whole number) in the human genome by PE-STOP or i-STOP strategy. The respective percentages are marked in each pie plot. *E*, the distribution of various types of base substitution in ClinVar database–recorded nonsense mutations is presented in a histogram. The number of records for each category of mutated alleles are marked on the columns. *F*, the coverages by PE-STOP or iSTOP for installing the ClinVar-documented pathogenic nonsense mutations are presented. The motif used for searching the iSTOP site is described in the text. The motif for PE-STOP was extended to including sequence from +1 to +9 after the cleavage site. The percentages of reachable and unreachable sites by PE-STOP and iSTOP are shown in the pie charts. PBS, primer-binding site; PE, prime editor; pegRNA, prime editing guide RNA; RTT, reverse transcription template; sgRNA, single-guide RNA.

The BEs and the more recent PEs represent an emerging generation of DSB/template DNA–independent precise genome editing platforms (23). BEs employ specific deaminase domains to enable targeted base transitions (Fig. S1*A*), whereas PEs harness an RT activity to install templated base conversions, small insertions, and small deletions (Fig. 1*A*). Because of the evident versatility of the PE platform, it holds strong application potentials. In principle, the basic PE system (PE2) consists of a single strand–nicking Cas9 (H840A) fused with an RT domain and a specialized pegRNA. The pegRNA contains a targeting sgRNA module and an extended 3′ region. This particular 3′ region features a primer-binding site (PBS, ∼13-nt) and an adjacent reverse transcription template (RTT) sequence encoding the intended small edits. Guided by the pegRNA to the target site, the nCas9 (H840A) module of PE would first make a nick on the noncomplementary strand at a position three nucleotides upstream of the “NGG” protospacer adjacent motif (PAM) motif. Subsequently, the 3′ PBS segment in the pegRNA would anneal with the sequence upstream of the nick. This would allow the adjacent RTT segment to direct the nCas9-fused RT domain for pegRNA-templated DNA synthesis. In consequence, the newly reverse-transcribed DNA may displace the original downstream DNA-end into a single-stranded 5′-flap structure (see Fig. S1*B* for illustration). Such an editing intermediate may be resolved by DNA repair mechanisms into a genetically modified first strand. Further cellular repair of the heteroduplex DNA at the target site may eventually lead to the complementary changes on the opposite stand (24). To promote the incorporation of the reverse-transcribed edits through this last repair stage, PE2 can be supplemented with an additional sgRNA (Fig. 1*A*), which in turn would nick the opposite strand and bias the cellular DNA repair system to accept the edits [PE3] (24).

An evident area of application for these newer editors is to empower precise inactivation of protein-coding genes. Of the established base editing–dependent gene inactivation tools, iSTOP enabled nonsense mutations at codons corresponding to only three amino acids because of its unitary activities for C-to-T conversion (12) (Fig. S1*A*), whereas the i-Silence selectively targeted the start codon (14). As PEs feature a much more diverse mutational scope than BEs (24), for the same purpose of causing gene inactivation, PE could be used to convert any codons corresponding to each of the 20 amino acids into a premature stop codon through single-, double-, or triple-base substitutions encoded by the pegRNA (Fig. S1*B*). We herein sought to develop such a tool that would be named as PE-STOP.

The designs of pegRNAs start with the selection of the target protospacers (flagged by the PAM) and the subsequent determination for the edit-encoding 3′-extension sequences. In practice, targeted nick by nCas9 on either the coding and noncoding strands can be considered for designing the 3′ extension sequence (PBS + RTT). The latter programs PE to install nucleotide changes downstream of the nCas9-initiated nick. A routine has been adopted by the field to refer to edits by their positions relative to the nick (*i.e.*, “+n”). For demonstration purposes (Fig. S1*B*), only the triple-base substitutions (a stop codon or its complement [XYZ] in place of an original codon) downstream of the nick are shown as examples. It is also worth noting that while PE may also be used for precise insertion of stop codons, the present work has focused on codon substitutions, for direct crossreference with the BE tools.

### The PE-STOP strategy is predicted to feature expanded genome coverages for gene inactivation and precise installation of nonsense mutations

Prior to examining the utility of PE-STOP in cells, we sought to first bioinformatically predict the overall coverages by PE-STOP (with NGG PAM) for targeting the genome space (Fig. 1*B*). The targeting scope by PE-STOP would also be compared with other DSB-independent precise gene-inactivating tools (*i.e.*, iSTOP and i-Silence).

#### Pipelines for bioinformatic determination of genome coverages by PE-STOP, iSTOP, and i-Silence

We downloaded the human reference genome (GRCh38) from the National Center for Biotechnology Information (NCBI) and collected the coding region sequences based on the annotation files (excluding pseudogenes and all noncoding RNAs). Next, the sequences were further extracted into arrays of coding exons, transcripts, or genes. The early exons in all human transcripts were further defined as n = 1/5 × total number of exons in a given transcript, rounded up to the next whole number (with the exon harboring the start codon defined as exon 1). The potential ORF sequences in the reference genome were obtained using the NCBI's ORFfinder software.

The genome-derived sequence arrays were next surveyed for target motifs characteristic to different tools. The coordinates of annotated start codons and stop codons were used to restrict hits within translated regions. Hits identified across exon junctions were excluded.

1. As PE shows higher efficiencies toward more proximal positions downstream of the nCas9 cleavage point (24, 36), we initially set a relatively conservative editing window that ranged from the +1 to +6 position (Fig. 1*B*). Such a parameter was followed for *in silico* analyses and for targeting applications throughout the study, unless otherwise indicated. Within this window, PE could potentially empower unlimited base substitutions. Therefore, the PE-STOP target motifs (from 5′ to 3′, with edit-conducive positions underlined) were set as “NN(15N)NNN*NGG*” and “*CCN*NNN(15N)NN,” two motifs flagged by the invariant strings (italic) representing the PAM element in the coding and complement sequences, respectively.
2. The C-to-T editing window for iSTOP is generally located from the fourth to eighth nucleotides in the nontargeted strand, in the downstream direction relative to the point corresponding to the 5′-end of an sgRNA (10). Therefore, the iSTOP target motifs (from 5′ to 3′, with the edit-conducive positions underlined and the target bases in *red*) were set as “NNNCAG(CAA/CGA)NN(11N)N*NGG*, NNNNCAG(CAA/CGA)NN(10N)N*NGG*, NNNNNCAG(CAA/CGA)NN(9N)N*NGG*, NNNNNNCAG(CAA/CGA)NN(8N)N*NGG*, or NNNNNNNCAG(CAA/CGA)NN(7N)N*NG*G” and “*CCN*N(11N)NNTGGNNN, *CCN*N(10N)NNTGGNNNN, *CCN*N(9N)NNTGGNNNNN, *CCN*N(8N)NNTGGNNNNNN, or *CCN*N(7N)NNTGGNNNNNNN,” two motif groups flagged by the invariant strings (italic) representing the PAM element in the coding and complement sequences, respectively. For iSTOP, an additional filter was placed to ensure the in-frame status (in relation to the annotated start codon) of the CAG/CAA/CGA/TGG codon within a hit.
3. The A-to-G editing window for i-Silence is generally located from the fourth to eighth nucleotides in the nontargeted strand, in the downstream direction relative to the point corresponding to the 5′-end of an sgRNA (11). Therefore, the i-Silence target motifs (from 5′ to 3′, with the edit-conducive positions underlined and the target bases in *red*) were set as “NNNATGNN(11N)N*NGG*, NNNNATGNN(10N)N*NGG*, NNNNNATGNN(9N)N*NGG*, NNNNNNATGNN(8N)N*NGG*, NNNNNNNATGNN(7N)N*NGG*” and “*CCN*N(11N)NNNATGNN, *CCN*N(10N)NNNATGNNN, *CCN*N(9N)NNNATGNNNN, *CCN*N(8N)NNNATGNNNNN, *CCN*N(7N)NNNATGNNNNNN,” two motif groups flagged by the invariant strings (*italic*) representing the PAM element in the coding and complement sequences, respectively. The sequence array corresponding to the first exon (*i.e.*, the one containing the start codon) of each transcript and gene was surveyed. An additional filter was placed to ensure that a hit should require the target nucleotides in the annotated start codons to fall within the edit-conducive positions.

#### Summaries of targetable genes, transcripts, exons, and potential ORFs by PE-STOP (*versus* the base-editing tools)

In our genome-wide survey of the GRCh38 assembly, a hit would be recorded if a given editor-specified target motif was identified within a record sequence as part of an array. When the CDS within every gene or every transcriptional isoform was considered as a potential target, the PE-STOP could achieve 100% coverages, enclosing the residual iSTOP misses and the apparent untargetable portions (∼42% genes and ∼57% transcripts) by i-Silence (Fig. S2, *A* and *B*). The suboptimal coverage by i-Silence (*versus* iSTOP) is consistent with previous determinations (14) and can be mainly attributed to the fixed locations of the target ATG codons.

Next, when the CDS region in every exon of a transcript was considered as a potential target, PE-STOP was predicted with 99.76% coverages in the human genome, compared with the 49.98% by iSTOP (Fig. 1*C*). As human genome may encode a large number of unannotated ORFs, the potentials for PE-STOP and iSTOP to install ORF-specific premature stop codons, and for i-Silence to target the putative start codons were considered. Here, PE-STOP also showed superior coverages over the BE-dependent tools for the collection of predicted ORFs (Fig. S2*C*). We also examined the potential coverages by PE-STOP on the CDS region of genes, transcripts, exons, and predicted ORFs in five other commonly targeted eukaryotic genomes (*Mus musculus* [GCF_000001635.27], *Danio rerio* [GCF_000002035.6], *Drosophila melanogaster* [GCF_000001215.4], *Caenorhabditis elegans* [GCA_000002985.3], and *Saccharomyces cerevisiae* [GCA_000146045.2]). Similar to the results with the human genome, PE-STOP were predicted to provide >99% coverage on every indicated category in these additional genome spaces (Fig. S3, *A*–*D*).

The apparently expanded coverage by PE-STOP in protein-coding regions propelled us to further consider its potential utility in functional studies. As nonsense mutations positioned within the early part of a transcript are more likely to lead to gene inactivation (12), we focused on the sequences corresponding to the early exons (n = 1/5 × total number of exons in a given transcript) in all human transcripts. The better theoretical coverages by PE-STOP (99.93%) than iSTOP (71.77%) within this trimmed coding space suggested the former’s potential as a more expansive loss-of-function tool (Fig. 1*D*).

#### Survey of ClinVar-documented nonsense mutations that may potentially be installed by PE-STOP

Nonsense mutations represent a major group of pathogenic variants. Importantly, besides the frequent consequences of gene inactivation, a significant fraction of such nonsense mutations was predicted to cause truncated protein isoforms with altered functionalities (22, 40). Therefore, precise modeling of nonsense variants in cell or humanized models is instrumental to study the pathogenic mechanisms and potential interventions. Here, we further examined the suitability of PE-STOP (or iSTOP) for installing the nonsense mutations presented in the ClinVar database (41).

The annotation file for ClinVar variants was downloaded from the NCBI database. Based on the mutation classifications, only the nonsense category of the single-nucleotide variants was selected. Such nonsense variants were also filtered for uniqueness by gene locations. The variants were further grouped based on the nature of nucleotide changes. The sequences flanking the mutations (100 bp) were extracted and were subjected to targeting queries by PE-STOP or iSTOP.

Because of the wide distribution of such disease variants in genes, we considered a less stringent (yet practical (24, 42)) editing window by PE-STOP (from +1 to +9; see Fig. 1*A* for comparisons) in the corresponding survey. Therefore, the target motif for PE-STOP (from 5′ to 3′, with the edit-conducive positions underlined) was set as “NN(15N)NNN*NGG*NNN” and “NNN*CCN*NNN(15N)NN”, two motifs flagged by the invariant strings (italic) representing the PAM element in the forward sequence and its complement, respectively. An additional filter was placed to ensure that a hit should require the target base (intended for substitution into the nonsense variant) to fall within the edit-conducive positions. On the other hand, as iSTOP only enables C to T (and G to A) base conversion, only sites corresponding to such subgroups of nonsense variants were subjected to queries with the iSTOP target motifs. Such motifs (from 5′ to 3′, with the edit-conducive positions underlined and the PAM-calling invariant strings italicized) were set as “NNNNNNNNN(10N)N*NGG*” for sites corresponding to C-to-T mutation and “*CCN*N(10N)NNNNNNNNN” for the G-to-A counterparts. The additional restriction was that a hit should require the target C or G to fall within the edit-conducive positions.

Based on such criteria, we surveyed all nonsense variants in the ClinVar database. Although many of them are indeed attributed to C-to-T or G-to-A mutations (Fig. 1*E*), iSTOP was only capable of introducing a subset of such mutations (15.9% of all nonsense variants) because of the activity window restraints (Fig. 1*F*, *lower*). In comparison, PE-STOP could potentially install a total of 30,851 (71.04%) ClinVar-documented nonsense variants (Fig. 1*F*, *upper*). Our analyses showed that PE-STOP could provide broad coverages for precisely installing disease-associated fixed-location nonsense mutations.

### General considerations for application of PE-STOP

In the seminal work that introduced the PE platform (24), a number of critical parameters for its application have been suggested. Following such an initial breakthrough, a number of contributions, including those from us, have also explored practical strategies to optimize prime editing (see (43) for a timely review). Herein, we adopt common reagents and typical parameters from the literatures in the development of PE-STOP method. The general application notes or recommendations are listed herewith.

1. In the present study, for direct comparisons with the BE tools, we would focus on applying PE-STOP method to installation of nonsense substitutions (with the number of nucleotides maintained).
2. Previous activity-screening experiments have established that PE exhibits higher efficiencies toward more proximal positions downstream of the nCas9-nicking point (24, 42). Therefore, for PE-STOP applications, we would survey for codon substitution sites located between +1 and +6 positions relative to an nCas9-dependent nick and prioritize the more proximal positions.
3. When the position for stop codon installation is not restricted (*e.g.*, for gene inactivation), multiple pegRNAs targeting different sites in the CDS would be screened for better efficiencies (26). The screen may be first carried out in an easily manipulatable cell line. Next, for generation of cell model for further studies, the best-performing pegRNA may be employed. On the other hand, when a fixed-location codon (*e.g.*, for making a precise truncational mutation) needs to be targeted, the proximity of an nCas9-nicking point upstream of the edited position should warrant strong considerations.

Additional note: A combinatorial codon-changing strategy toward simultaneous installation of 2x or 3x adjacent stop codons may potentiate the performances by PE-STOP (see the later Results section on application of PE-STOP at the genomic loci).

4. It is worth noting that the sizes for PBS and RTT affect PE efficiencies (24, 42). To simply the initial testing, we elected to use a fixed rule for the sizes of PBS (13-nt) and RTT (with a ∼14-nt extension following the encoded edits) in different pegRNAs. Further tuning of the PBS/RTT lengths may be implemented, if the initial testing does not identify a pegRNA with adequate activity.
5. To condition high prime editing efficiencies, the improvement strategies based on the use of an optimized PE construct (PEmax (37)), and on pegRNA stabilization with the exoribonuclease-resistant RNA motif (35), were simultaneously employed for our entire study. The latter strategy entails the use of an “xr-pegRNA” construct expressing a pegRNA joined by a ∼80-nt xrRNA stabilizing motif at the 3′ end (see Experimental procedures section).
6. Further inclusion of a nicking sgRNA to target the opposite strand at a position 50 to 90 bp away from the pegRNA-directed nick is needed to potentiate editing (in PE3 format).
7. For transfection in a 24-well format, a total of 1.3 μg plasmids (including 900 ng PEmax, 300 ng xr-pegRNA, and 100 ng of the corresponding nicking sgRNA) would be transfected per well. Cells can be harvested after 72 h (or 48 h for editing of a reporter plasmid).
8. When targeting a given genomic locus, it is recommended to use a pegRNA construct with a fluorescent marker (*e.g.*, enhanced GFP [EGFP]). Sorting of the transfectants by fluorescence-activated cell sorting (FACS) would enrich the edited cells.
9. FACS sorting after transfection may facilitate the establishment of edited single-clone cell lines poised for downstream experiments.

### PE-STOP mediates nonsense substitutions of various codons in a plasmid reporter

To test the applicability of PE-STOP, we constructed a reporter plasmid encoding a double-fluorescent mRuby-EGFP fusion protein. It was conceivable that introduction of a premature stop codon within EGFP would result in the expression of a truncated reporter species lacking the functional EGFP part. Since premature termination mutation within an intron-less mRNA generally does not signal nonsense-mediated mRNA decay (44), editing by PE-STOP may cause selective reduction of EGFP fluorescence in the cells.

**Figure 2:**
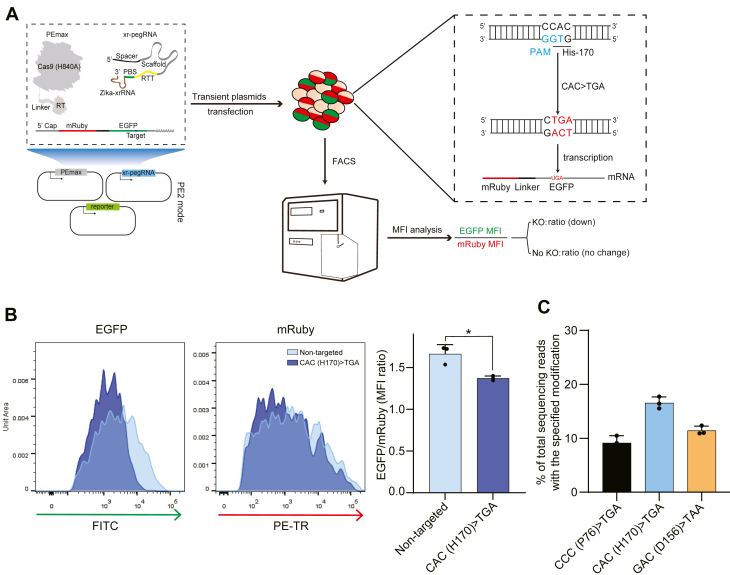
**PE-STOP mediates nonsense substitutions of various codons in a plasmid reporter.***A*, the general workflow for testing the effect by PE-STOP on a reporter system is presented. The *left scheme* shows the editing components and the reporter. Note the presence of xrRNA motif on the pegRNA (xr-pegRNA), a configuration used throughout the present study. An mRuby-EGFP fusion construction was adopted as the reporter system. The components of PE-STOP including xr-pegRNA and PEmax can be cotransfected with the reporter plasmid. The installation of a premature stop codon in the EGFP region would conceivably lead to selective downregulation of EGFP (*versus* mRuby) fluorescence. The sequence information of the PE-STOP-targeted codon (His170) in the reporter is shown in a zoomed-in fashion. The PAM sequence is marked in *blue*. The target codon is *underlined*. The intended stop codon is shown in *red*. *B*, the components of PE-STOP (PE2) including xr-pegRNA and PEmax were cotransfected with the reporter plasmid (30 ng) into HEK293T cells. The levels of EGFP and mRuby fluorescence in response to PE-STOP action are shown in the representative histograms. Here, the PE-STOP was programmed to mutate a histidine codon in EGFP (“CAC (H170) > TGA”). Cells transfected with a nontargeting sgRNA were used as a control (“nontargeted”). On the *right*, the ratios between median fluorescence intensities of EGFP and mRuby (“EGFP/mRuby”) further indicate the selective downregulation of EGFP levels. Data were obtained from three biological replicates (shown as the mean ± SD) and were analyzed by Student’s *t* tests (∗*p* < 0.05). *C*, editing efficiencies by PE-STOP (PE2) for targeting three different codons in the EGFP-coding region are shown. PCR amplicons corresponding to the edited regions were analyzed by targeted deep sequencing. The reads with the perfect edits were specified to evaluate the editing efficiencies (n = 3 biological replicates). Data are presented as mean values ± SD. EGFP, enhanced GFP; HEK293T, human embryonic kidney 293T cell line; pegRNA, prime editing guide RNA; xrRNA, exoribonuclease-resistant RNA.

We next designed a pegRNA to install a nonsense mutation at a codon of CAC (corresponding to histidine-170) to TGA (Fig. 2*A*), a modification apparently beyond the scope by iSTOP. The adoption of the xrRNA-joined pegRNA framework (used throughout the present study) is also indicated in the illustration. Next, the plasmids for PEmax and pegRNA (or a nontargeting sgRNA) were cotransfected as in a PE2 format, together with the reporter plasmid, into the human embryonic kidney (HEK) 293T cells. The cells were subjected to flow cytometry 48 h after transfection (Figs. 2*A* and S4*A*). Fluorescence histograms from the mRuby^+^EGFP^+^ cells showed that PE-STOP targeting led to a more substantial downregulation of EGFP levels compared with the changes in mRuby levels (Fig. 2*B*), consistent with our initial assumption. Indeed, determinations of the ratio between the quantitated levels of EGFP and mRuby (median fluorescence intensities) also demonstrated the particular dampening of EGFP levels by PE-STOP. These promising observations prompted us to design two more pegRNAs targeting respectively at a codon of CCC (corresponding to proline-76) and a codon of GAC (corresponding to aspartate-156) in EGFP (Fig. S4*B*). In addition, an extra set of controls for each targeting group were prepared by replacing PEmax with an RT-less H840A nCas9 that would allow DNA targeting without installing nonsense substitutions. Following transfection of HEK293T cells with individual site-specific PE-STOP plasmids and the reporter, the DNA samples were harvested for targeted next-generation sequencing (NGS) analyses. In parallel, the levels of EGFP in the transfected cells were examined by fluorescence microscopy and immunoblotting. The NGS results showed that all three PE-STOP groups enabled similarly scaled moderate levels (∼10–20%) of nonsense mutations in the reporter (Fig. 2*C*). In addition, all three PE-STOP groups demonstrated notable decreases in EGFP levels in comparison to the nontargeted group as well as to their corresponding nonmutational nCas9 groups (Fig. S4, *C* and *D*). It is worth noting that nCas9 targeting to the reporter alone modestly reduced the levels of EGFP (*versus* the nontargeted control). We reasoned that such background effect by nCas9 targeting *per se* might be responsible for the seemingly amplified PE-STOP-associated changes in EGFP protein signals, in relation to the sequencing results (see comparisons between Figs. 2*C* and S4, *C* and *D*). The potential effects by nCas9- or PE-mediated targeting on reporter mRNA expression were also tested. The results showed that the levels of reporter mRNA expression remained similar among control-, nCas9-, or PE-targeted cells (Fig. S4*E*). Collectively, our results with the reporter demonstrated the feasibility by PE-STOP to install stop codons and to drive reductions in target gene expression.

### PE-STOP allows efficient nonsense substitutions within genomic contexts

**Figure 3:**
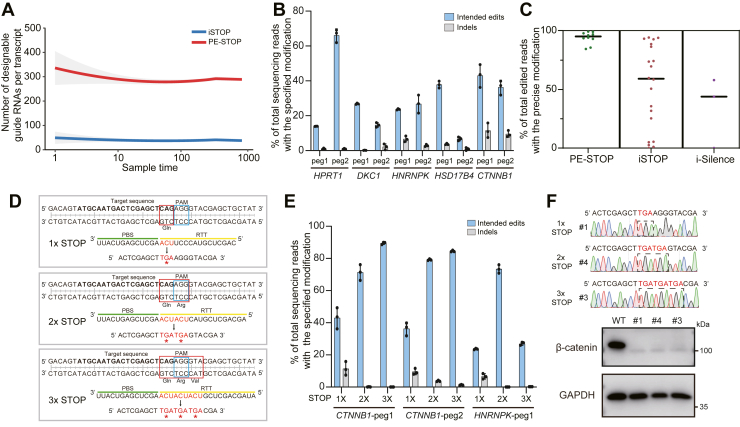
**PE-STOP allows efficient nonsense substitutions within genomic contexts.***A*, the graph shows the predicted numbers of stop codons programmable by pegRNAs (PE-STOP, *red*) or sgRNAs (iSTOP, *blue*) on an average human transcript. A given number of transcripts were randomly sampled for three times and were used to model the overall numbers of editable sites *via* locally estimated scatterplot smoothing. *B*, the editing efficiencies by PE-STOP for targeting five genes (two sites/gene) are shown. The editing efficiencies and the associated indel levels were determined from the deep sequencing data (n = 3 biological replicates). Data are presented as mean values ± SD. *C*, the on-target editing purities by PE-STOP, iSTOP, and i-Silence are presented. The precise editing efficiencies are indicated by the percentages of reads harboring only the targeted mutations (perfect edits) within all edited reads. Each data point shown in the scattered plot represents the averaged value (from three biological replicates) for a single site. The *horizontal lines* in each editing group represent the median levels of precisions for each editing tool. *D*, as an example, the design of PE-STOP for installing single (1x), double (2x), or triple (3x) stop codons in *CTNNB1* (peg1 site) is illustrated by the layouts of target and pegRNA sequences. The PAM motifs are highlighted by the *blue boxes*, whereas the targeted codons are highlighted by the *red boxes*. The PBS (*green line*) and RTT *(yellow line*) sequences within respective pegRNAs for installing 1x, 2x, and 3x stop codons are shown, with the edits marked in *red*. *E*, the efficiencies and the associated indel levels by PE-STOP for installing single (1x), double (2x), or triple (3x) stop codons at three different sites (within two genes) are shown. For installing various numbers of stop codons, the pegRNAs featured the same spacer and PBS sequences but different RTT sequences encoding the edits. Data were obtained from three biological replicates (shown as the mean ± SD). *F*, HEK293T cells were transfected with PE-STOP plasmids to program single, double, or triple stop codons in the gene encoding β-catenin (*CTNNB1*, “peg1” site as in [*D* and *E*]). Following transfection, single clones were cultivated for subsequent determination of genotypes. The Sanger sequencing results from clones corresponding to the homozygous mutants with various forms of nonsense mutations (1× STOP, 2× STOP, and 3× STOP) are presented on the *top*. The levels of protein expression for β-catenin in cells derived from the WT and mutant clones were determined by Western blot, with the use of GAPDH as loading control (*lower*). HEK293T, human embryonic kidney 293T cell line; PBS, primer binding site; pegRNA, prime editing guide RNA; RTT, reverse transcription template; sgRNA, single guide RNA.

Encouraged by the evident activities of PE-STOP in the reporter system, we sought to investigate its performances in targeting endogenous genes. In correlation with the trend from earlier genome-wide survey (Fig. 1), bioinformatic analyses on a given number (up to 1000) of random transcripts and subsequent data fitting (locally estimated scatterplot smoothing) demonstrated that an average number of >300 PE-STOP pegRNAs could be designed for the CDS of each transcript, a coverage fivefold higher than that by iSTOP (NGG PAM considered in both cases) (Fig. 3*A*). We next pursued editing of five individual genes (*HPRT1*, *CTNNB1*, *HNRNPK*, *HSD17B4*, and *DKC1*) with PE-STOP. Herein, for each of the five genes, two PE-STOP pegRNAs and the corresponding nicking sgRNAs were designed (Fig. S5). As discussed in aforementioned general methodology section, a fixed rule was adopted here regarding the sizes for PBS (13-nt) and RTT (with a ∼14-nt extension following the encoded edits) in different pegRNAs. Next, the plasmids for PEmax, together with those for the specific sets of pegRNAs and nicking sgRNAs, were cotransfected into HEK293T cells. About 72 h after transfections, the EGFP^+^ (marker for the pegRNA construct) cells were sorted by FACS, which was followed by preparation of genomic DNA and targeted PCR amplification. The PCR products were then subjected to NGS analyses. Overall, these targeting attempts at different sites led to various levels of stop codon installation (median efficiency of 29%, ranging between 6% and 66%) (Fig. 3*B*). For comparison purposes, we also carried out editing experiments on the same five genes with the iSTOP strategy (each with multiple sgRNAs) and on the three of these genes with targetable start codons (those of *DKC1*, *HPRT1*, and *HSD17B4*) with the i-Silence strategy (Fig. S5). The results showed that in general, iSTOP and i-Silence led to more efficient installation of desired mutations, with respective median efficiencies at 45% (3–92%) and 71% (36–93%) (Fig. S6, *A* and *B*). Despite the relative higher efficiencies, these two BE-dependent approaches were associated with significant degrees of bystander editing (Fig. S6*C*), as readily expected (10, 11, 45). Potentially, the bystander editing by iSTOP would become a particular concern for studying nonsense mutants with gain of functions, as it may induce additional mutations. With the aforementioned target sites considered together, iSTOP and i-Silence, respectively, showed median levels of 52% and 33% in precision, whereas PE-STOP yielded a much higher median level (∼93%) of editing purity (Fig. 3*C*). Collectively, the significantly improved sequence coverages and on-target precisions by PE-STOP over the base-editing tools implicated the broad application potential of PE-STOP for investigations of natural nonsense mutations and for finer structure–function studies on given proteins.

The improved sequence coverages by PE-STOP would allow more extensive screening of target sites to offset the less consistent PE efficiencies (43), given that the goal of application is to empower site-independent gene loss of function. Indeed, in an independent screening effort to target four different genes with various numbers of pegRNAs (8 for *ALDOB*, 4 for *PRNP*, 2 for *RNF2*, and 1 for *RIT1*, with each pegRNA programmed for mutating a codon corresponding to a different amino acid), the gene-wise top efficiencies were >45% for three of four genes (up to 58%, 45%, and 67% for *ALDOB*, *PRNP*, and *RIT1*, respectively) (Fig. S7). It is straightforward to carry out such pegRNA-screening experiments in an easily manipulatable cell line, for example, the HEK293T cell line employed here. Such screening efforts would eventually facilitate the application of PE-STOP (adopting a high-activity pegRNA) in a functionally relevant cell model.

An additional regard for improving PE-STOP performances might entail the control of DNA mismatch repair (MMR) pathway that was found to primarily act as a checkpoint to reject mismatched editing intermediates and to undesirably invoke formation of indels (despite their relatively low levels in PE) (37, 46). Indeed, combining PE with inhibition of MMR pathway formulated a new version of PE tool (PE4/5) featuring better efficiencies and higher fidelities (37). Importantly, as the MMR pathway prefers small-sized mismatches, deliberately expanding the size of the edits (from ∼1–3 nt to ∼6–10 nt) has been implemented as a convenient alternative strategy to bypass this editing checkpoint (37, 47). The latter developments inspired us to set up PE-STOP for installing moderately enlarged edits. To this end, we subjected PE-STOP to targeting adjacent codons toward two (2x) or three (3x) consecutive stop codons in HEK293T cells (Figs. 3*D* and S8*A*). Here, four genes (*CTNNB1*, *HNRNPK*, *DKC1*, and *HPRT1*) were targeted at a total of six independent sites (Figs. 3*E* and S8*B*). The deep-sequencing analyses of transiently transfected cells demonstrated that the 2x and 3x codon-targeting PE-STOP overall led to noticeably higher levels (2.3- and 2.2-fold at the median levels) of editing than their single-codon counterparts (Fig. S8*C*). Consistent with previous observations upon inhibition of MMR (37, 47), the better efficiencies in 2x and 3x codon-combined targeting were also associated with generally higher editing purities (Fig. S8, *D* and *E*). Taken together, these results suggested the potential of designing combinatorial codon changes for enhanced PE-STOP performances.

Given the aforementioned investigations on the application parameters of PE-STOP, we set to validate the potential by PE-STOP for generation of gene-inactivated cell models. Initial experiments on PE-STOP-transfected cells (with *CTNNB1* and *HNRNPK* as respective targets, sorted on EGFP fluorescence) showed evident decreases in the levels of the corresponding proteins (β-catenin and heterogeneous nuclear ribonuceloprotein K) (Fig. S9, “pegRNA 1” sets in Fig. 3*B* used for both targets). However, as such downregulation of protein expression might only be attributed in part to the direct installation of a premature stop codon (see NGS results in Fig. 3*B* as references), we considered the need to establish stable clonal populations following transfections. Subsequently, cells were transfected with the respective sets of PE-STOP for installation of single, double, and triple stop codons in *CTNNB1* (illustrated in Fig. 3*D* and in reference to the “peg1” site in Fig. 3*E*). The transfectants were subjected to single-clone cultivation. Judged by the results from Sanger sequencing, each of the three targeting groups yielded a homozygous mutant clone (1 of 16, 1 of 8, and 1 of 10, respectively) (chromatograms in Fig. 3*F*, *top*). Importantly, the protein samples from the obtained homozygous mutant clones showed apparent deficiencies in β-catenin protein expression (Fig. 3*F*, *bottom*). Furthermore, we carried out PE-STOP applications targeting three genes at a total of four sites in mouse N2a cells. The NGS results from the transient transfectants demonstrated a median of 25% efficiency for stop codon installation, with all groups exhibiting high editing purities (Fig. S10, *A* and *B*). Following single clonal cultivation of *Pdcd1*-targeted transfectants (corresponding to the “peg1” group in Fig. S10*A*), a homozygous mutant clone (1 of 13) could be established with the aid of Sanger sequencing (Fig. S10*C*).

We also applied PE-STOP for targeting the stably expressed SV40 large T antigen (encoded by a genomic fragment, *SV40gp6*) in HEK293T cells. The *SV40gp6* transgene had been originally introduced into the parental HEK293 cells (to establish the HEK293T cells) in 1980s (48). Initial investigations had shown that this transgene in the HEK293T cells contributed to increasing the yield of recombinant viral vectors (49). Nevertheless, recent works instead challenged such a role by this transgene, conceivably because of long-term genetic drifting of HEK293T cells (50, 51). We reasoned that PE-STOP-mediated editing of *SV40gp6* in HEK293T cells may help to clarify such uncertainty. Five pegRNAs against the two exons within the integrated viral sequence were designed (Fig. S11, *A* and *B*). We later focused on the use of “peg3” (favorable editing efficiency and purity at 27% and 97%, respectively) for establishment of monoclonal lines. A clone (#7) with nonsense mutation at *SV40gp6* was identified by Sanger sequencing (Fig. S11*C*). The results of Western blot further confirmed the absence of SV40 large T protein in this clone (Fig. S11*D*). A parallelly isolated WT clone (#5) that showed similar growth characteristics as the mutant clone (#7) was adopted as the control. These cells were next tested for packaging capacities for recombinant lentiviral vectors. Following transfection of the transfer plasmid (with EGFP as the insert) and the packaging constructs to the WT and mutant cell lines, the yielded viral titers were determined (Fig. S11, *E* and *F*). Compared with the WT control, the *SV40gp6* nonsense-mutated cells produced only slightly reduced titers (1.59× *versus* 1.34 × 10^6^ units/ml) of the transduction-competent lentiviral vector (Fig. S11*G*). These results confirmed the nonessential role of large T antigen for lentiviral vector production in the present-day HEK293T cultures (51). Altogether, the aforementioned series of data demonstrated the applicability of PE-STOP as a versatile and an effective tool for precise installation of nonsense mutations and for generation of gene-inactivated cell models.

### PE-STOP-induced nonsense mutations may be subjected to subsequent precise reversions by the same PE platform

For cell-based genetic studies, in addition to generation of a mutant model, subsequent studies on the gene-rescued cells would be critical for definitive assignments of genotype–phenotype relationships (52). The rescue experiments would help to exclude the artifacts originated from nonspecific targeting or genetic drifting of the cell model. The possibility of establishing a second reverse mutation in gene-edited cells would provide unique opportunities for defining gene functions. Such a reverse mutation experiment may become more advantageous in studying specific gain-of-function mutations, when confirmatory targeting of alternative sites (with a different guide RNA) may not be applicable. Moreover, it would be instrumental to ensure high levels of precision for the reverse mutation, as a full functional rescue may require perfect restoration of the WT CDS. In this regard, the PE platform may be suited to install reverse mutations owing to its versatility and precision. Such a scenario of reverse mutation could be exemplified in a reporter assay where a prospective PE-STOP-installed nonsense mutation was rescued by PE to enable expression of the full-length reporter marked by green fluorescence (Fig. S12*A*).

**Figure 4:**
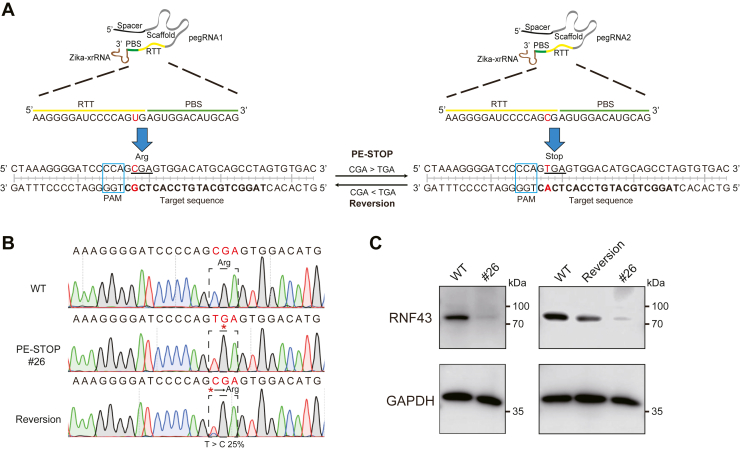
**PE-STOP-induced nonsense mutations may be subjected to subsequent precise reversions by the same PE platform.***A*, the design of PE for successive nonsense and reverse mutation is illustrated by the layouts of target sequences (in *RNF43*) and the corresponding pegRNAs. The sequences from the WT and the nonsense mutant allele (R519X) are placed on the *left* and *right sides*, respectively. The pegRNA1 is designed to install the nonsense mutation, whereas the pegRNA2 is adopted to enable the reverse mutation. The RTT (*yellow line*) and PBS (*green line*) sequences within respective pegRNAs are shown. On the WT and mutated (R519X) sequences, the PAM motifs are highlighted by the *blue boxes*, and the targeted codons are *underlined*. *B*, HEK293T cells were first transfected with PE-STOP (in PE3 format) to install R519X mutation. Following sorting the transfected cells into single clones, a clone (#26) with homozygous nonsense mutation was identified by Sanger sequencing (*middle panel*). The cells derived from this clone were transfected again with PE to drive the reverse mutation. The transfected cells were pooled by fluorescent marker sorting. This mixed population of cells were further expanded in culture for 7 days. The DNA samples were harvested. Visible levels of reverse mutation can be captured by Sanger sequencing (*bottom panel*). *C*, the protein samples were harvested from cells derived from the WT clone and the nonsense mutant clone (#26). Following transfection of the mutant cells with the back-mutation PE plasmids for 48 h, the transfectants were harvested *via* FACS. The cells were further expanded for 7 days. The total protein samples were harvested. The indicated protein samples were analyzed by WB. FACS, fluorescence-activated cell sorting; HEK293T, human embryonic kidney 293T cell line; PBS, primer binding site; PE, prime editor; pegRNA, prime editing guide RNA; RTT, reverse transcription template; WB, Western blot.

To formally introduce such a two-stage mutation-reversion strategy, we next focused on a naturally occurring nonsense mutation (at Arg519) of a tumor suppressor gene, *RNF43* (53). The pegRNA1 would be designed to first convert the codon CGA (R519) into the stop codon TGA. Subsequently, the mutant cells would then be introduced with the pegRNA2 for reversing the codon TGA (X519) back into the original codon CGA (Fig. 4*A*). Given that the specific edit herein would spare the PAM motif, the pegRNA1 and pegRNA2 would target at the same location and respectively enable WT and mutant allele–selective editing. Generally speaking, a same nicking sgRNA would be conveniently adopted in the separate PE3 mix for the nonsense mutation and the rescue. In the ensuing experiments, we subjected HEK293T cells to successive applications of PE-STOP and reverse mutation. Following the initial introduction of PE-STOP (with pegRNA1), single clones of cells were screened. A clone (#26) was validated by Sanger sequencing to harbor homozygous R519X mutation in *RNF43* (Fig. 4*B*). The homozygous mutation was correlated with a strong diminishment of RNF43 protein levels (Fig. 4*C*, *left*). Thereafter, the cells derived from this mutant clone were retransfected with the PE3 components (with pegRNA2) to program the reverse mutation. The transfected cells were harvested in a pool *via* fluorescent marker–based sorting. In this mixed population, a fraction (∼25%) of the TGA allele was reversed to the original CGA genotype (Fig. 4*B*). Importantly, such a partial degree of precise “X519R” reverse mutation enabled apparent re-expression of RNF43 protein (Fig. 4*C*, *right*). Although future precise manipulation of *RNF43* in more relevant cell models (*i.e.*, colon cancer cell lines (53)) are warranted, these early stage results in the HEK293T cell model have clearly shown the feasibility of programming successive point mutation and reversion using PE.

It is conceivable that a large portion of stop codons installed by PE-STOP are potentially reversible by a second round of PE through the use of the same PAM motif, given that the fifth and sixth positions (Gs in the NGG motif) remain intact in the nonsense-mutated genotype (see in the aforementioned *RNF43* example). On the other hand, reverse mutations of iSTOP-installed stop codons would only be achieved by a different editor (*e.g.*, by an ABE or PE) whose suitability would be subjected to independent restraints of available activity windows. The reverse editing by ABE is likely to also induce bystander base changes, which may inadvertently yield a non-WT genotype associated with altered functions. To theoretically infer the applicability of PE and BE tools for enabling nonsense mutation and subsequent reversion, we performed coverage analyses (Fig. S12*B*), tailoring the bioinformatic queries used earlier (Fig. 1*B*) with additional filters. The results demonstrated that 100% of the human transcripts (records) can potentially be manipulated by PE through sequential nonsense and precise reverse mutations. On the other hand, although nearly 100% of human transcripts may also be sequentially targeted for nonsense and back mutations (respectively, *via* iSTOP and ABE platforms), about 59% of the transcripts would be not suitable for precise back mutations (following iSTOP), because of the bystander activities of ABE (Fig. S12*B*, *red box*). Furthermore, given the restraints in activity windows, about 30% of the transcripts may even not be amenable to successive nonsense and reverse mutations *via* the order of iSTOP and PE (Fig. S12*B*, *blue box*). These analyses implicated an additional advantage of PE-STOP (compared with iSTOP), in the compatibility of its product mutations with a subsequent round of precise rescue by PE. Practically, the same PE protein (*e.g.*, delivered stably) could be used readily for sequential rounds of mutations. Taken together, such a prospect by PE to install successive nonsense and reverse mutations provide a promising strategy for definitive genotype–phenotype investigations.

### PE-STOP exhibited better specificity profiles than iSTOP and i-Silence

**Figure 5:**
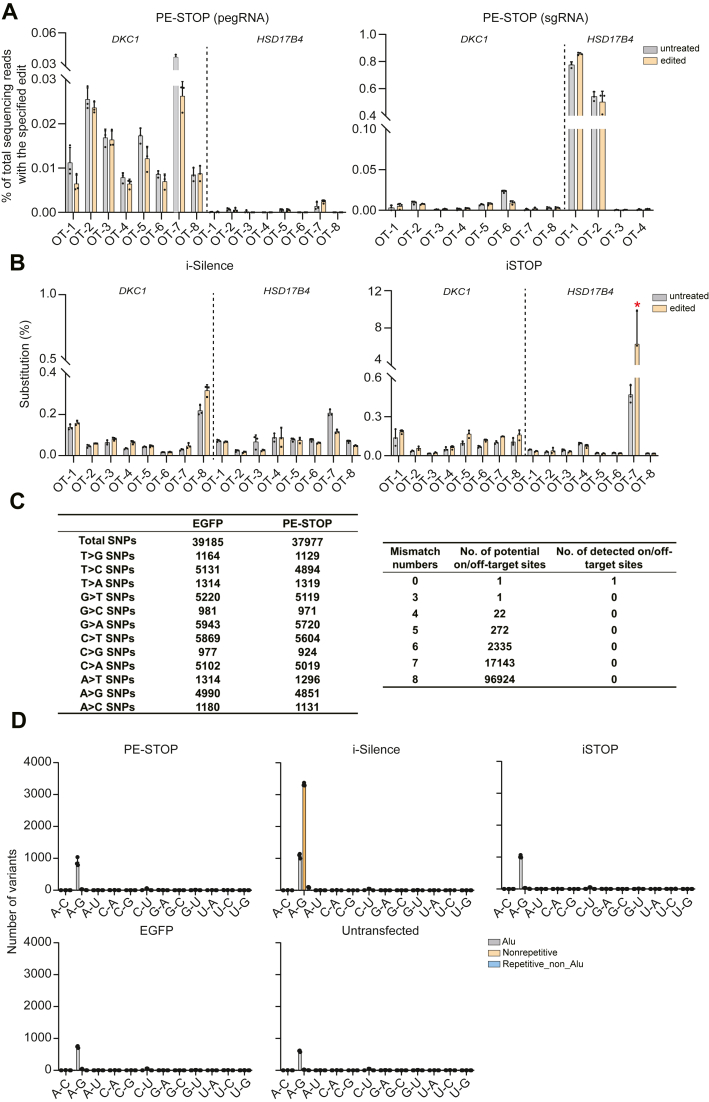
**PE-STOP exhibited better specificity profiles than iSTOP and i-Silence.***A*, DNA off-target (OT) analyses were performed following the application of PE-STOP against *DKC1* (peg1) and *HSD17B4* (peg1). Targeted deep sequencing experiments were performed for the pegRNA (*left*)- and nick sgRNA (*right*)-associated potential OT sites. The OT sites analyzed here were a collection of high-score sites predicted by the Cas -OFFinder program. Samples from untreated cells were used as controls. The levels of sequence variances at these sites are presented in graphs (with indications of their corresponded pegRNAs [target genes]). The average values from three biological replicates are shown (±SD). *B*, DNA OT analyses were performed following the application of i-Silence (*left*) and iSTOP (*right*) against *DKC1* (sgRNA1) and *HSD17B4* (sgRNA1). Targeted deep sequencing experiments were performed for the sgRNA-associated potential OT sites. Samples from untreated cells were used as controls. The levels of base substitutions corresponding to ABE or CBE activities at these sites are presented in graphs (with indications of their corresponded pegRNAs [target genes]). The average values from three biological replicates are shown (±SD). An apparent indication of iSTOP-dependent OT editing (C-to-T) is highlighted by a *red mark*. *C*, WGS analyses were performed following the application of PE-STOP against *HSD17B4* (peg1). The EGFP-transfected group was used for comparisons. The reads from untransfected cells were used to filter out the basal genetic variances in both groups. In addition, sites with 0 to 8 mismatches from the targeted site were queried for edits. On the *left*, the numbers of *de novo* SNPs in these two groups are displayed for indicated substitution types. The results on the *right* show identification of only the desired edit by WGS. *D*, potential editing by different editors on cellular RNA were assessed by analyses of RNA-Seq data. Numbers of all RNA sequence variants with base substitutions were determined in samples from cells introduced with different editors (PE-STOP [PEmax + pegRNA + nick sgRNA], iSTOP [AncBE4max + sgRNA], and i-Silence [ABE8e + sgRNA]). Samples from the untransfected and EGFP-transfected cells were also used for comparisons. The numbers of variants are grouped in accordance with indicated repetitive or nonrepetitive features. The average values from three biological replicates are shown (±SD). ABE, adenine base editor; CBE, cytosine base editor; EGFP, enhanced GFP; pegRNA, prime editing guide RNA; sgRNA, single guide RNA; WGS, whole-genome sequencing.

Encouraged by the apparent versatility and validated effectiveness of PE-STOP, we next examined its editing fidelity (*versus* iSTOP and i-Silence) by targeted deep sequencing. HEK293T cells were transfected with PE-STOP (in PE3 format), iSTOP, or i-Silence to target two endogenous genes (*DKC1* and *HSD17B4*). Eight high-score predicted OT sites for each targeting experiment (corresponding to the guide RNAs used) were selected for targeted analyses. In addition, for PE-STOP-harnessed nicking sgRNAs against *DKC1* and *HSD17B4*, eight and four potential OT sites were analyzed, respectively. The results showed that the application of PE-STOP did not cause editing at these potential pegRNA- or nick-sgRNA-related OT loci (Fig. 5*A*). For the BE-dependent tools, while the application of i-Silence did not induce changes at the profiled OT sites (Fig. 5*B*, *left*), the use of iSTOP led to apparent OT editing (C-to-T) at one of the analyzed OT sites (>5% of the reads) (Fig. 5*B*, *right*). It is also worth noting that iSTOP might potentially incur additional guide RNA–independent editing on DNA (21, 54, 55), presenting a further specificity concern for its application.

For a more comprehensive assessment of editing fidelity by PE-STOP, whole-genome sequencing (WGS) was subsequently performed using genomic DNA from mock- (EGFP) or PE-STOP-transfected HEK293T cells (targeting *HSD17B4*). Reads from the two transfected groups were compared with those from untransfected cells, which led to the calling of overall similar numbers of baseline SNPs in the mock transfection and the PE-STOP groups (Fig. 5*C*, *left*). Next, potential editing at sites with up to eight mismatches from the targeted sequence were selected for further analyses. Importantly, no edits other than the on-target modification by PE-STOP were identified from such WGS analyses (Fig. 5*C*, *right*). Collectively, consistent with the observations from previous studies (24, 31, 32), our results demonstrated that the current PE-dependent tool (PE-STOP) featured minimal OTs in the genome.

For the BEs, another source of OTing is represented by the deaminase-dependent promiscuous base-modifying activities toward cellular RNAs (56, 57). Therefore, we further extended the comparisons between PE-STOP and the BE-dependent tools to their potential off-targeting activities in RNAs. To this end, RNA-Seq analyses were carried out on the samples from PE-STOP-, iSTOP-, and i-Silence-transfected cells. Variants in RNA sequences were called *via* the RADAR pipeline established previously (58). The data showed that in comparison to the untransfected control and the mock transfection (EGFP) groups, the PE-STOP samples did not show significant changes in the counts of single nucleotide variants in RNAs (Fig. 5*D*). In contrast, i-Silence (based on ABE8e (57)) introduced A to G mutations at a substantial number (>3000) of sites in nonrepetitive RNA sequences (Fig. 5*D*), consistent with previous studies (56, 57). Our results further showed that iSTOP (based on AncBE4max (59)) resulted in a small number (∼50) of C-to-U-modified sites in RNAs (Fig. 5*D*), in line with the low RNA off-targeting activities for this specific form of deaminase (58, 60).

Genome editor-triggered DNA damage response may indirectly select for cells deficient in core components safeguarding genome integrity (4, 5). Therefore, an editing tool’s intrinsic characteristics in triggering a DNA damage response might also impact its specificity profiles. As γ-H2AX represents a commonly used marker for DNA damage signaling, we promptly analyzed its levels in HEK293T cells transfected with PE-STOP or other editing tools (all targeting *HPRT1* gene, programmed with the same guide RNA sequence except for the i-Silence group). Indeed, an evident upregulation of γ-H2AX could be observed in the positive control group (Cas9/sgRNA transfectants). The iSTOP and i-Silence groups were associated with milder degrees of γ-H2AX induction, correlated to their adoption of a less damaging Cas9 nickase. Interestingly, the PE-STOP groups appeared to feature further reduced engagement of γ-H2AX signaling, compared with the aforementioned two groups (Fig. S13). It is also worth mentioning that the PE-STOP (PE3) and iSTOP groups analyzed here featured equivalent levels of editing efficiencies in our earlier experiments (see *HRPT1* subgroups in Figs. 3*B* and S6 [the top-activity set in either graph]). These results on DNA damage signaling are likely to reflect the differences among these platforms regarding editing intermediates and/or byproducts. Taken together, this series of specificity-related analyses have strongly suggested a lower undesirable mutational risk associated with the application of PE-STOP.

## Discussion

Premature stop codons resulting from either frameshift or substitutional mutations severely affect gene functions (61). The ability to install nonsense mutations with high coverages in cell or animal models would significantly expedite our studies regarding gene function and regulation as well as the mechanisms underlying genetic diseases and cancers. Conventionally, nonsense mutations may be installed *via* CRISPR/Cas9-depedent generation of DSB followed by subsequent error-prone or DNA template–dependent repair, resulting in frameshift or precise mutations (2, 3). However, such DSB-based editing approaches generate complexed genotypes and can cause edit-independent complications (23). PEs represent a very recent generation of DSB-independent genome editing tools that have shown strong potentials for basic research and translational applications. In this study, we adapt PE into a universal tool for precise installation of nonsense mutations (PE-STOP).

The present development of PE-STOP extends previously established DSB-independent base-editing gene inactivation tools (*e.g.*, iSTOP (18) and i-Silence (14)). Distinct from such BE tools, PE-STOP is not limited by the types of the codons targeted for nonsense mutation, given their presence within an available activity window. Indeed, compared with iSTOP and i-Silence, PE-STOP potentially has more expansive coverages (near 100%) over the genome space for targeting genes, transcripts, exons, and predicted ORFs (Figs. 1 and S2). In addition, for more localized mutagenesis such as for targeting early exons to cause gene inactivation, and especially for installing clinically relevant nonsense mutations, PE-STOP is predicted to provide markedly higher coverages than iSTOP (Fig. 1). Such broad coverages by PE-STOP highlight its strong potential not only for inactivation of specific coding regions but also for precise establishment of truncational mutations. The bioinformatic target-mining pipelines established here would also contribute to the future construction of high-throughput targeting libraries for screening purposes.

Our work adopted several latest developments aimed at enhancing PE activities (35, 37). Equipped with such upgrades, in multisite targeting trials, PE-STOP generally resulted in ∼25 to 30% editing efficiencies in HEK293T cells and N2a cells. Such targeting efficiency *via* transient transfections enabled subsequent generation of gene-inactivated single clones lacking the expression of β-catenin, large T antigen (HEK293T), and with validated homozygous mutation in *Pdcd1* [N2a] (Figs. 3, S10, and S11). We demonstrated that it is also possible to use pegRNA screening to achieve more potent targeting (Fig. S7). Another evident advantage by PE-STOP lies in its high editing purity, while bystander editing remains largely unavoidable by BEs (Fig. 3*C*). This favorable feature further supports the application of PE-STOP in studies of nonsense mutants with likely gain of functions, which may be compromised by the neighboring bystander mutations. It is relevant to note that although recent developments of newer versions of BEs have improved the precisions and/or genome coverages for this family of tools (23), the intrinsic limitations of BE for nonsense-substituting only selected codons still exist.

Moreover, we found that the design of pegRNAs to program combinatorial changes of adjacent codons might improve the editing efficiencies (Fig. 3*E*), likely *via* bypassing an MMR-dependent editing checkpoint (37, 47). While comprehensive assessments under more diverse application contexts are still warranted for such enhancement strategy, these results have pointed to a promising prospect that further developments shall refine PE-STOP into an increasingly efficient tool. To facilitate the interested readers to carry out PE-STOP experiments, we have listed a number of application notes in the “General considerations for application of PE-STOP” part in the Results section.

In gene-edited cell models, a proper rescue of gene activities would be essential for accurately assigning phenotypes to mutations (52). In this regard, a direct reverse mutation at the very genomic locus would provide an ideal strategy for gene rescue. Here, we further demonstrate that the versatility of PE may be harnessed for successively establishing nonsense and reverse mutations (Fig. 4). For the most part, such reverse mutations would readily target the same PAM motif (NGG, from fourth to sixth nt after the cleavage point), given the maintenance of the “GG” upon the primary mutations. Indeed, bioinformatic survey confirm that PE-STOP-installed stop codons are more amenable to subsequent rescue by PE, compared with a parallel scenario with iSTOP-established stop codons whose rescue would require a different platform (Fig. S12). Given such unique potential of PE for successive rounds of primary and reverse mutations, such a strategy may be broadly applied in the future to define genotype–phenotype relationships in cells. It is worth noting that as the cells may adapt to genetic changes through multistage mechanisms (62), the outcomes by reverse mutations may be cell context dependent. In this regard, this proposed toolkit for primary and reverse mutations may provide a window into identifying new mechanisms underlying the control of cell states.

To establish the specificity profiles for PE-STOP in reference to the previous base-editing tools, we carried out targeted deep sequencing, WGS, and RNA sequencing/variant calling in cells introduced with various editors (Fig. 5). Indeed, we validated the superior fidelity profile of PE-STOP over iSTOP and i-Silence. Overall, given its versatility, precision, and specificity, PE-STOP may represent an important tool for investigating nonsense mutations in focused analyses as well as in high-content screening studies. Because of the strong versatilities of the PE technology, the PE-STOP established here only reflect a specific area within its full scope of useful applications. We envision that future development of PE technology shall hold great promise to broadly impact basic research and translational studies.

## Experimental procedures

### Plasmid construction

PEmax (catalog no.: L174820) and pU6-sgRNA-GFP (catalog no.: 85451) plasmids were purchased from Addgene. The U6-driven pegRNA expression construct that contained a 3′-xrRNA motif (pU6-xr-pegRNA-GFP) was described previously (35). For incorporation of different pegRNAs into the vector, we first used paired primers (xr-pegRNA-bone-F: AGCTAGGTCTCCTGTCAGGCCTGCTAGTCAGCCACAGTTTGG, xr-pegRNA-bone-R: TCTCTCGGTCTCACGGTGTTTCGTCCTTTCCAC) to amplify the backbone using pU6-xr-pegRNA-GFP as the template with the aid of 2× Phanta Flash Master Mix (Dye Plus) (Vazyme). The plasmid backbone was then cut by BsaI-HFv2 (NEB) for overhangs (top strand with ends of 5′ TGTC, bottom strand with 5′ CGGT overhang). The guide RNA scaffold sequence was synthesized as long oligos directly (Table S1). For spacer oligos, the top strand oligo featured 5′ ACCG and 3′ GTTTC ends, whereas the bottom strand oligo comprises a 5′ CTCTGAAAC end (to condition 5′ ACCG and CTCT overhangs on the top and bottom strands, respectively) (Table S2). The xr-pegRNA 3′ extension, including the PBS sequences and RTT sequences, was also synthesized (top strand oligo with 5′ GTGC overhang and the bottom strand oligo with 5′ GACA overhang) (Table S2). Based on the plasmid construction methods described in our previous study (35), all the components of xr-pegRNA were assembled by DNA ligase Solution Ⅰ (DNA Ligation Kit, version 2.1; Takara) according to the manufacturer′s protocols. We elected to use a fixed rule for designing the sizes for PBS (13-nt) and RTT (with a ∼14-nt extension following the encoded edits and generally avoiding the base “c” as the closest base to the guide RNA scaffold) in different pegRNAs, for simplicity purposes. For nick sgRNA, the scaffold sequence was synthesized as long oligos (Table S1), and the spacer was synthesized (with 5′ ACCG overhang on the top strand and 5′ CTCT overhang on the bottom strand). The subsequent construction workflow was the same as previously described (26).

### Cell culture and transfection

HEK293T (American Type Culture Collection; CRL-3216) and Neuro-2a (N2a, American Type Culture Collection; HTB-96) cells were cultured in 10 cm dishes with Dulbecco's modified Eagle's medium (GIBCO) supplemented with 10% fetal calf serum (v/v) (Gemini) and 1% penicillin–streptomycin at 37 °C with 5% CO_2_. Cells were routinely passaged at a ratio of 1:3 at 90% confluency by digesting with 0.25% pancreatin (containing EDTA). During transfection, cells were cultured in 24-well plates in three biological replicates and transfected with 1.3 μg plasmids (including 900 ng PEmax, 300 ng xr-pegRNA, and 100 ng corresponding nick sgRNA) per well, when cells reached an approximate 70% to 90% confluency. Transfections were carried out with the aid of EZ *Trans* (Life-iLab; catalog no.: AC04L091) reagent and according to the manufacturer′s protocols. For experiments involving WGS or RNA-Seq, HEK293T cells were cultured in larger scale (10 cm dishes). Subsequently, 13 μg of total plasmids for PE-STOP (9 μg PEmax, 3 μg xr-pegRNA, and 1 μg corresponding nick sgRNA), 12 μg total plasmids for iSTOP or i-Silence (9 μg AncBE4max or ABE8e with 3 μg corresponding sgRNA), and 5 μg total plasmids for CTRL group (5 μg pCMV-GFP) per dishes were used for transfections. Cells were transfected at approximately 60% to 90% confluency.

### Cell sorting and analysis by flow cytometry

For FACS analyses, cells were harvested 48 h after transfection. For sorting of cells by FACS to enrich edited cells, cells were harvested 72 h after transfection. For analyses of the reporter system, a total of 10,000 cells were recorded. The results were analyzed by the use of FlowJo software (BD, version 10). For analyzing the editing of endogenous genes, a total of 5000∼10,000 GFP^+^ cells were collected for each sample (BD Aria III). To obtain single-cell colonies, the transfected cells were sorted into batches of single cells (Moflo Astrios EQ). Sorted cells were cultured in a 96-well plate. The cells were harvested to examine the mutational status after 7∼10 days in culture.

### Genomic DNA extraction

After allowing genome editing to occur after transfection, the GFP^+^ (for PE) or mCherry^+^ (for ABE and CBE) cells were sorted by FACS and collected in tubes containing 300 μl of PBS. Following transfer of the cells and further centrifugation at 1000*g* for 2 min, the cell pellets were harvested and added with 40 μl QuickExtract DNA Extraction Solution (Lucigen). The genomic DNA samples were prepared according to manufacturer′s instructions.

### Sanger sequencing and targeted deep sequencing

The isolated genomic DNA samples were amplified *via* PCR with Phanta Max Super-Fidelity DNA Polymerase (Vazyme) to obtain target fragments for Sanger sequencing. For deep-sequencing experiments, we first used paired primers with specific barcodes (each corresponding to a given experimental condition) to amplify the target sequence. The amplified DNA products were purified with the DNA Recovery Kit (AXYGEN) according to the manufacturer′s instructions. For each forward or reverse primer used in amplifying sequence, 5-nt barcodes were added to the 5′ end of these primers. The specific primer sequences are listed in Table S3. OT site prediction was performed using the Cas-OFFinder tools (63) (http://www.rgenome.net/cas-offinder). The high-score sites chosen for analyses are listed in Tables S4 and S5. Deep-sequencing analyses were performed on samples with a sequencing depth of more than 50,000 high-quality reads per amplicon. The BWA (64) and Samtools (65) were employed for mapping the pair-ended reads to the human reference genome (GRCh38). The Fastq-multx (66) was employed for demultiplexing the reads. The CRISPResso2 tool was used for determining the editing efficiencies and levels of precision (67). The prime editing efficiency was calculated as percentage of reads with the desired edit that do not contain indels within total mapped reads. The ABE/CBE editing efficiency was also calculated as percentage of reads with the desired edit and no indels within total mapped reads. The purity of PE was calculated as percentage of precise edits within total reads with desired edits regardless of copresence of indels. For ABE/CBE, precise editing efficiency was calculated as percentage of reads with edits at the target codon only within total edited reads. The corresponding imprecise editing rate was calculated as percentage of reads with desired edits along with bystander mutations within total edited reads. All results were derived from analyses of three biological replicates.

### Western blot

Radioimmunoprecipitation assay lysis buffer supplemented with the Halt protease & phosphatase inhibiter (Thermo Scientific; catalog no.: 78442) was used to obtain total proteins from cultured cells. Equal amounts of total proteins (20 μg) were separated on SDS-polyacrylamide gels and transferred onto a polyvinylidene fluoride membrane (0.45 μm). The membrane was then incubated with primary antibodies diluted 1:1000 after protein transfer. Afterward, secondary antibodies (diluted 1:15,000) were incubated on the membranes. The images were captured with an Amersham Imager 600 (GE Healthcare). The antibodies used are listed in Table S6. For transiently transfected cells, 48 h after cell transfection, 50,000 marker-positive cells were sorted by FACS. Cells were further cultured in 6 cm dishes for another 7 days prior to their harvest for protein analyses.

### WGS analysis

For WGS, 500,000 transfected cells (GFP^+^ for PE and mock-transfected groups) were sorted by FACS (Moflo Astrios EQ). The genomic DNA samples were prepared using phenol–chloroform extraction. WGS libraries were prepared using standard protocols for the Novaseq S2 platform at the Anoroad Genome Institute. A total of 90 Gb WGS data of human HEK293T cells for each sample were generated. The WGS OT analyses was performed using a previously described pipeline (68). All SNP annotation files were download from NCBI, UCSC, and 1000 Genome Project (1000G) websites. Clean reads were aligned to human reference genome GRCh38.p14 assembly with BWA using default parameters (64). Duplicated reads were removed with Picard (http://broadinstitute.github.io/picard/). After sorting and duplicating the BAM file (65), the GATK suite (69) was used for base quality score recalibration. The following processes were employed to identify reliable *de novo* SNPs. (i) GATK, LoFreq (70) and Strelka (71) were used to identify SNPs separately with their default parameters. (ii) Background variants in untransfected human 293T cells beyond the SNP records from dbSNP and USCS repeat regions were filtered out. (iii) VCF files were filtered for depth <10 and alleles frequency <10% to filter out imprecise SNPs. (iv) SNPs that could be identified by all three different callers (GATK, LoFreq, and Strelka2) were considered as high-confidence *de novo* SNPs. Finally, all 12 types of base conversion SNPs were separated for display.

### RNA-Seq and variant calling

For RNA-Seq, 500,000 transfected cells (GFP^+^ for PE and mock transfection groups and mCherry^+^ for ABE and CBE groups) were sorted by FACS (Moflo Astrios EQ). The TRIZOL reagent (Vazyme) was used to extract total RNA. The RNA-Seq libraries were prepared according to standard protocols for the NovaSeq platform and subjected to commercial RNA-Seq services (Anoroad Genome Institute). All SNP annotation files were downloaded from NCBI, UCSC, 1000G, and Exome Sequencing Project websites. The RNA OT analysis was called by the following pipeline (58). (i) Clean read was aligned to human reference genome GRCh38.p14 assembly using the HISAT2 (72) and BWA programs. rRNA was removed, and duplicated reads were marked by the Picard tool. Unique reads with up to six mismatches were selected for further analyses. (ii) The GATK tool was used for SNP calling. The SNPs from dbSNP, 1000G, and ESP were excluded as baseline variants. Low-quality SNPs were called based on the following criteria: mapped reads <2, hits per billion-mapped-bases <3, or editing ratio <0.05 and were subsequently removed. (iii) All 12 types of base conversions within Alu repeats, non-Alu repetitive sequences, and nonrepetitive regions were determined for graphic presentations.

### Analysis of lentivirus titer

The lentiviral transfer plasmid featured a cytomegalovirus promoter–driven EGFP. For examining whether the packaging ability of HEK293T cells might be affected by the absence of large T antigen, the gene-inactivated cells were subjected to comparisons with their WT counterparts. To this end, the WT (#5) and KO (#7) HEK293T cells were first seeded on six-well plates at 6.5 × 10^5^ cells per well, respectively. Cells were transfected with 3 μg plasmids (including 1.2 μg transfer plasmid, 800 ng pMD2.G [Addgene, catalog no.: 12259], and 1 μg psPAX2 [Addgene; catalog no.: 12260]) 12 to 16 h after seeding (at 70–90% confluency). The plasmids were mixed with 9 μl EZ *Trans* (Life-iLab; catalog no.: AC04L091) and added to the cells. After 6 to 8 h transfection, the cells were refed with fresh medium. The supernatants were collected 48 h after transfection. After filtered through 0.45 μm filters, the stocks were stored at −80 °C. For determination of vector titers in the prepared stocks, they were used to transduce a common batch of parental HEK293T cells. These cells were seeded on 24-well plates at 87,000 cells per well in 500 μl culture medium and were subsequently added with 2, 4, 8, 12 of the lentiviral vector–containing supernatants. The culture medium was replaced with fresh medium after 12 h transduction. After further cultivation for 48 h, cells were harvested for analysis of the GFP^+^ ratio by FACS (LSRFortessa X20). After linear fitting of EGFP^+^ cell numbers to the volume of supernatants, the vector titers were determined (transduction units/ml).

### Determination of potential genome-wide coverages by PE-STOP, iSTOP, and i-Silence

The sequence and annotation files for multiple genomes were downloaded from NCBI. The calculation of genome coverage with different codon-changing strategies was performed by the following pipeline: (i) Extracting coding regions based on the annotation files and removing the non-mRNA and pesudo gene sequences for the respective coverage queries on genes, transcripts, and exons. (ii) Using ORFfinder in NCBI (set as the default parameters) to extract potential ORF sequences for coverage queries on predicted ORFs. (iii) Composing motif files based on different editing strategies. The composed motifs in (iii) would be used to search editable sites in CDSs (i) and ORF sequences (ii). The coordinates of exon–exon junctions were used as a parameter in the search program to filter out the erroneous “cross-exon” motif hits. The motifs corresponding to PE-STOP, iSTOP, and i-Silence are detailed in the texts related to Figure 1*B*. In practice, the start codons were first located in the genome space and used to define the reading frame. For i-Silence hits, queries were made to determine whether the i-Silence motifs might cover a start codon. For iSTOP and PE-STOP hits, queries were made to determine whether their respective motifs would be found within a sequence in question. For iSTOP, an additional restriction was made to ensure the in-frame status for the convertible codon in a potential hit. For analyses of disease-associated mutations, the data corresponding to the nonsense mutation variants were first downloaded from the ClinVar. The annotation information was used to enumerate variants within each group. After setting the coordinates for these mutation-affected sites, a similar query strategy as aforementioned was applied to determine their potential coverages by PE-STOP or iSTOP. Here, the editing window of PE-STOP was set as +1 to +9 bp. To establish potential coverages by an editor (PE) or editor combinations (*e.g.*, CBE + ABE) to target human transcripts for successive rounds of primary and reverse mutations, a similar pipeline involving step (i) and (iii) were employed, except for the use of more complex motifs corresponding to both primary and reverse edits (Table S7). All codes used in this part have been deposited in GitHub (https://github.com/huang-0323/PE-stop-finder).

### Quantitative PCR analysis

For analyzing the mRNA expression of mRuby-EGFP reporter, the total RNA from the cells was harvested 48 h after transfection (RNA isolater Total RNA Extraction Reagent; Vazyme). The complementary DNA synthesis was performed using the HiScript Q RT SuperMix for quantitative PCR (qPCR) (+gDNA wiper) (Vazyme) and random hexamers as primers. The qPCRs were set up using AceQ qPCR SYBR Green Master Mix from Vazyme. Two primer pairs were designed to amplify the 5′ and 3' positions of the reporter gene. The relative mRNA levels were determined using the 2-ΔΔCt method. The primer sequences are listed in Table S8.

### Statistical information

All individual data points presented except for the WGS data were summarized results from three biological replicates. Analyses and graphing were carried out with SPSS (IBM, version 23) and GraphPad Prism (version 8), respectively. Data are presented as means ± SD as indicated in the legends. In box plots, the center line shows the medians and the box limits correspond to upper lower quartiles, whereas the whiskers mark the largest and smallest points. Statistical significance of differences between two groups was determined using Student’s *t* tests (unpaired).

## Data availability

The WGS, RNA-Seq, and deep-sequence data have been deposited in the NCBI SRA under Bioproject number PRJNA916653 (https://dataview.ncbi.nlm.nih.gov/object/PRJNA916653?reviewer=p99lqeg6420feepvsnsbubhqdk). The reference human genome assembly GRCh38/hg38 used for read mapping is an openly accessible resource (https://www.ncbi.nlm.nih.gov/assembly/GCF_000001405.40).

## Code availability

Codes are deposited at the GitHub repository (https://github.com/huang-0323/PE-stop-finder).

## Supporting information

This article contains supporting information.

## Conflict of interest

The authors declare that they have no conflicts of interest with the contents of this article.
