# Supplementary material for: PE-STOP: A versatile tool for installing nonsense substitutions amenable for precise reversion
Source: J Biol Chem. 2023 Jun 19;299(8):104942. doi: 10.1016/j.jbc.2023.104942 (PMC10365944; doi:10.1016/j.jbc.2023.104942)
Supplement: Supporting Figures S1–S13 [file mmc1.docx]

**Supplementary information**

**Contents:**

**Supplementary Figures and Legends (S1-S13)**

**Supplementary Tables (see a separate “.xls” file)**

Table S1 Scaffold sequence information used for pegRNA and sgRNA.

Table S2 Sequence information of pegRNAs and sgRNAs used in this study.

Table S3 Primers used for deep sequencing in this study.

Table S4 Potential off-target sites analyzed in *HSD17B4*- and *DKC1*-edited cells (by PE-STOP).

Table S5 Potential off-target sites analyzed in *HSD17B4*- and *DKC1*-edited cells (by iSTOP and i-Silence).

Table S6 Antibodies used in this study.

Table S7 The motif sequences used to determine coverages by different editor combinations for successive primary and back mutations in genome space.

Table S8 Primers used for qPCR in this study

**
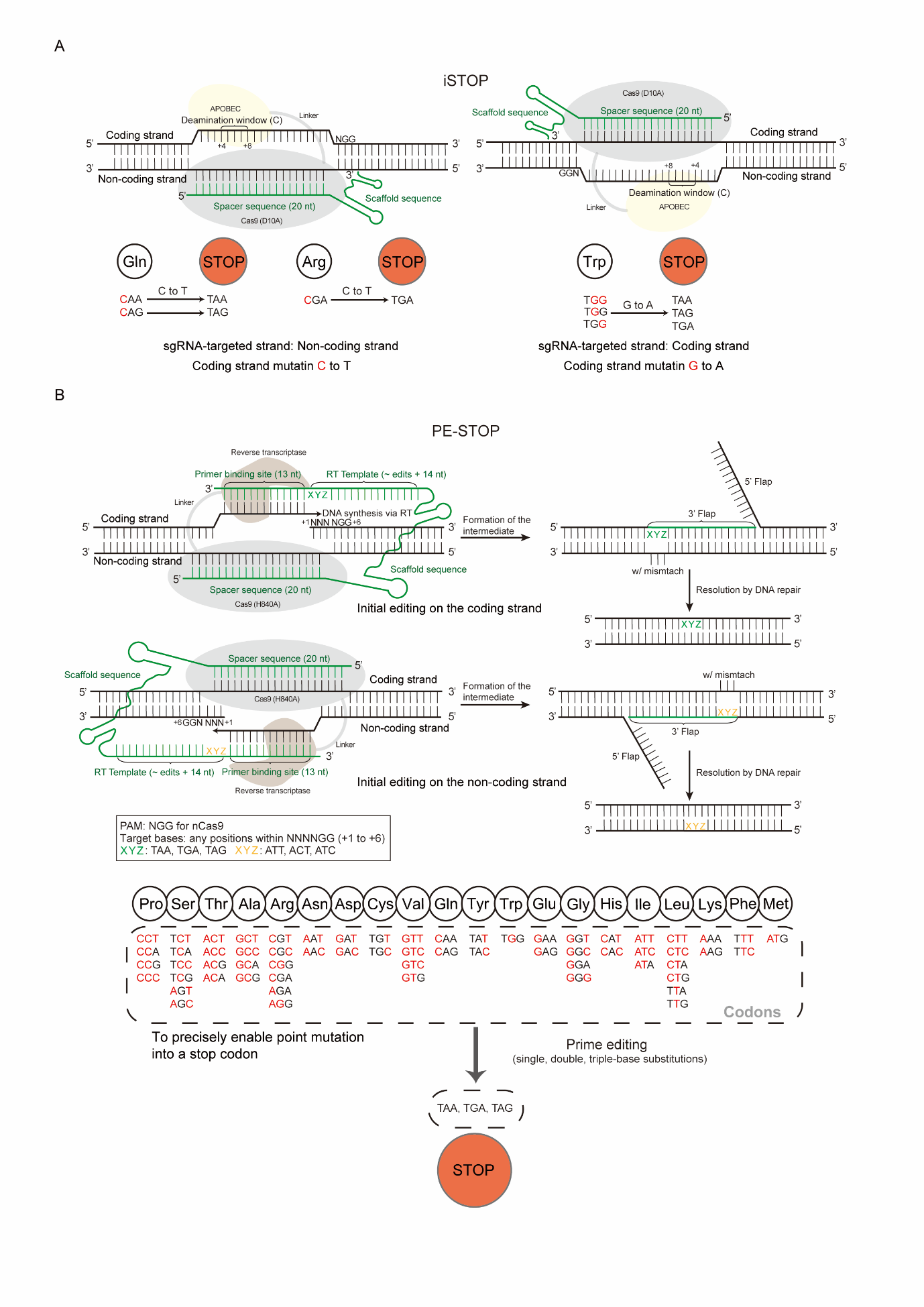
**

**Figure S1: The potential for iSTOP and PE-STOP to mutate codons corresponding to different types of amino acids into stop codons.**

1. The schematic diagram shows the potential of CBE for installing nonsense mutation (iSTOP). The construction of CBE is based on the mutant Cas9 (D10A) deficient in DSB induction. The incorporation of a cytidine deaminase activity to the Cas9 part forms the core architecture of CBE that enables C-to-T and G-to-A substitutions within an editing window at the guide RNA-targeted site. Therefore, iSTOP can drive conversion of several different types of properly located codons into stop codons (TAA/TAG/TGA).
2. The schematic diagram shows the potential of using PE to install stop codons (PE-STOP). The nCas9 (H840A) and reverse transcriptase motifs of PE are indicated. The nicked target DNA is depicted, with the cleavage site (denoted as +1 position) and PAM sequence (NGG, ending at the +6 position) indicated. The pegRNA molecule is shown in green. The segments corresponding to the spacer, scaffold, RTT and PBS are respectively marked. The “XYZ” within the RTT represents the programmed edits for installing a stop codon. In practice, each editing by PE-STOP would correspond to 1-3 concomitant base changes, dependent on the nature of the targeted codon. Reverse transcription based on the RTT sequence in pegRNA leads to the formation of an editing intermediate, containing both a newly synthesized 3′ flap and a 5′ flap originated from the nick. The DNA repair mechanisms would resolve such an intermediate to either accept or reject the edits. In the former case, a nonsense mutation would be installed. As PE shows higher activities toward sites more proximal to the nick, an editing window of from +1 to +6 is used for the present study (unless otherwise indicated). In this context, PE-STOP can enable any 1-3 base substitution at the editing window (+1 to +6), potentially capable of converting unlimited types of codons into stop codons.

**
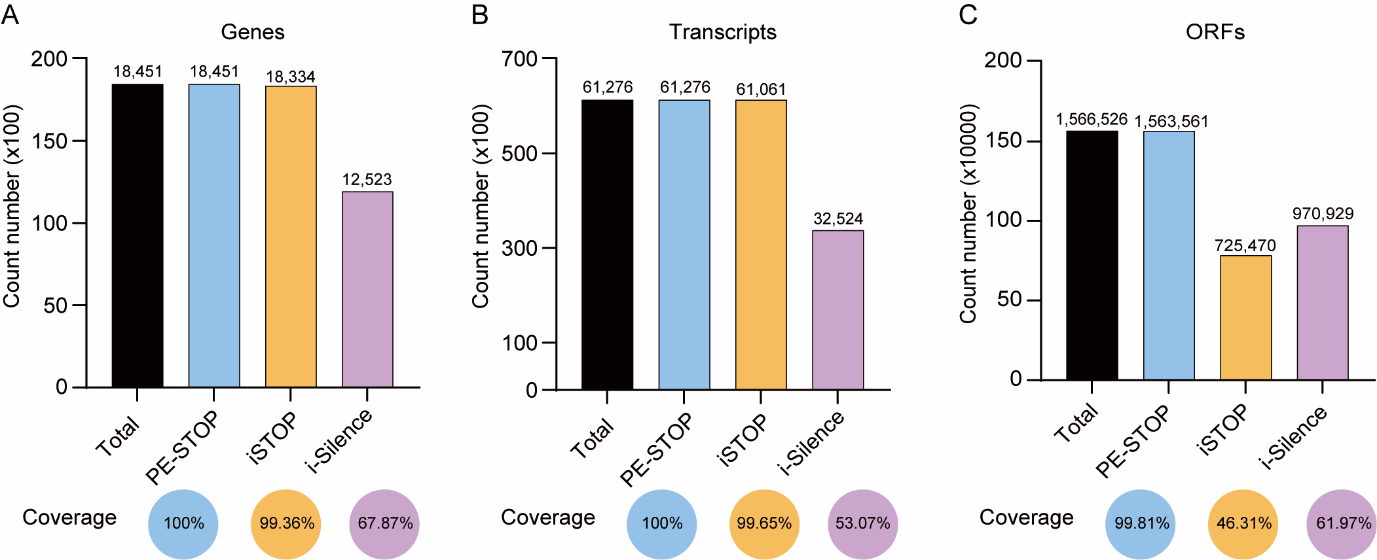
**

**Figure S2: The percentages of genes, transcripts and predicted ORFs that may be targeted by different strategies for nonsense mutations.**

a-C. The bar graphs shows the number of genes (A), transcripts (B) and ORFs (C) in the human genome potentially targetable by PE-STOP, iSTOP or i-Silence. A hit would be recorded when a target gene/transcript/ORF is targetable with at least one guide RNA. The numbers are marked above each column, while the coverage percentages are indicated under each column.

**
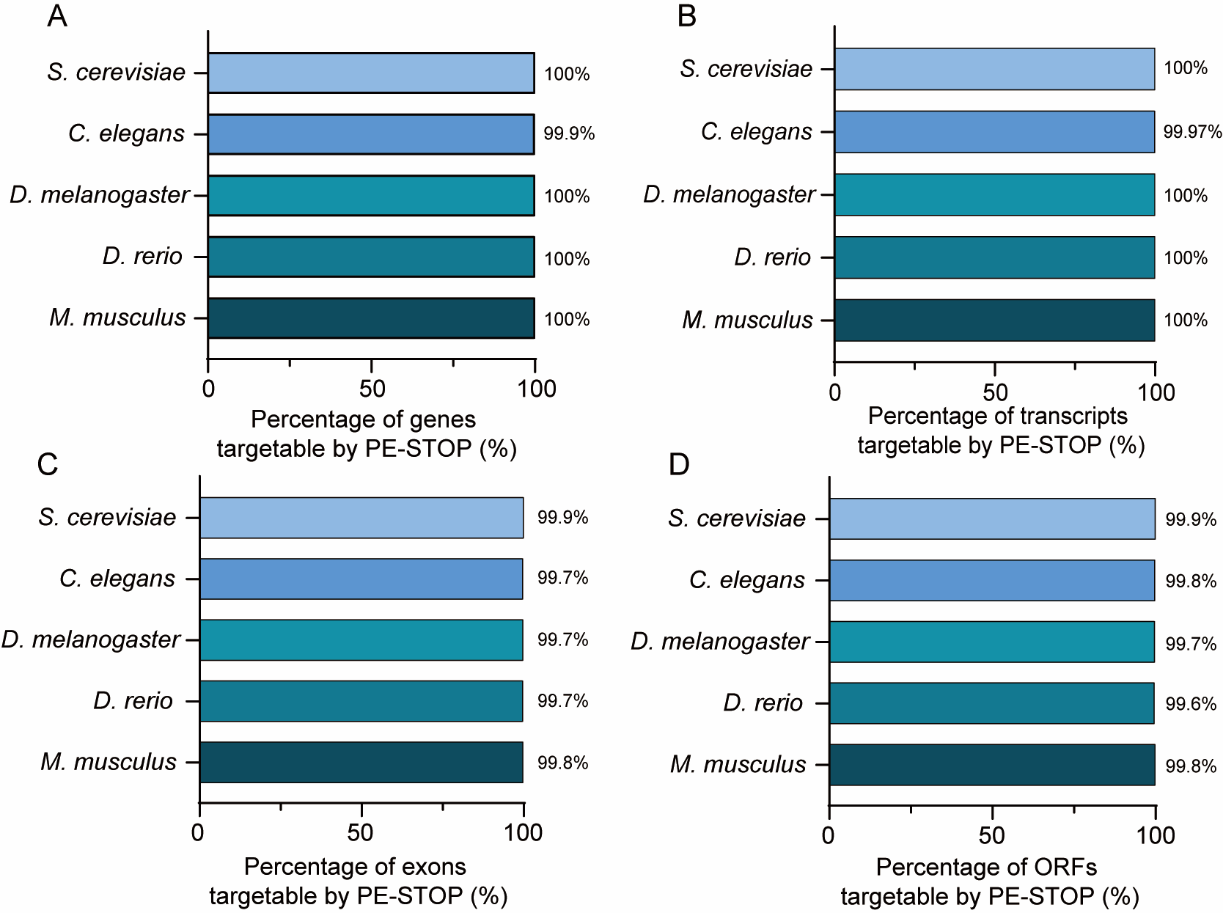
**

**Figure S3: The coverages by PE-STOP for genes, transcripts, exons and predicted ORFs in different eukaryotic species.**

A-D. Percentages of genes (A), transcripts (B), exons (C) and predicted ORFs (D) potentially targetable by PE-STOP in five eukaryotic species are shown. The percentages are marked near each data bar.

**
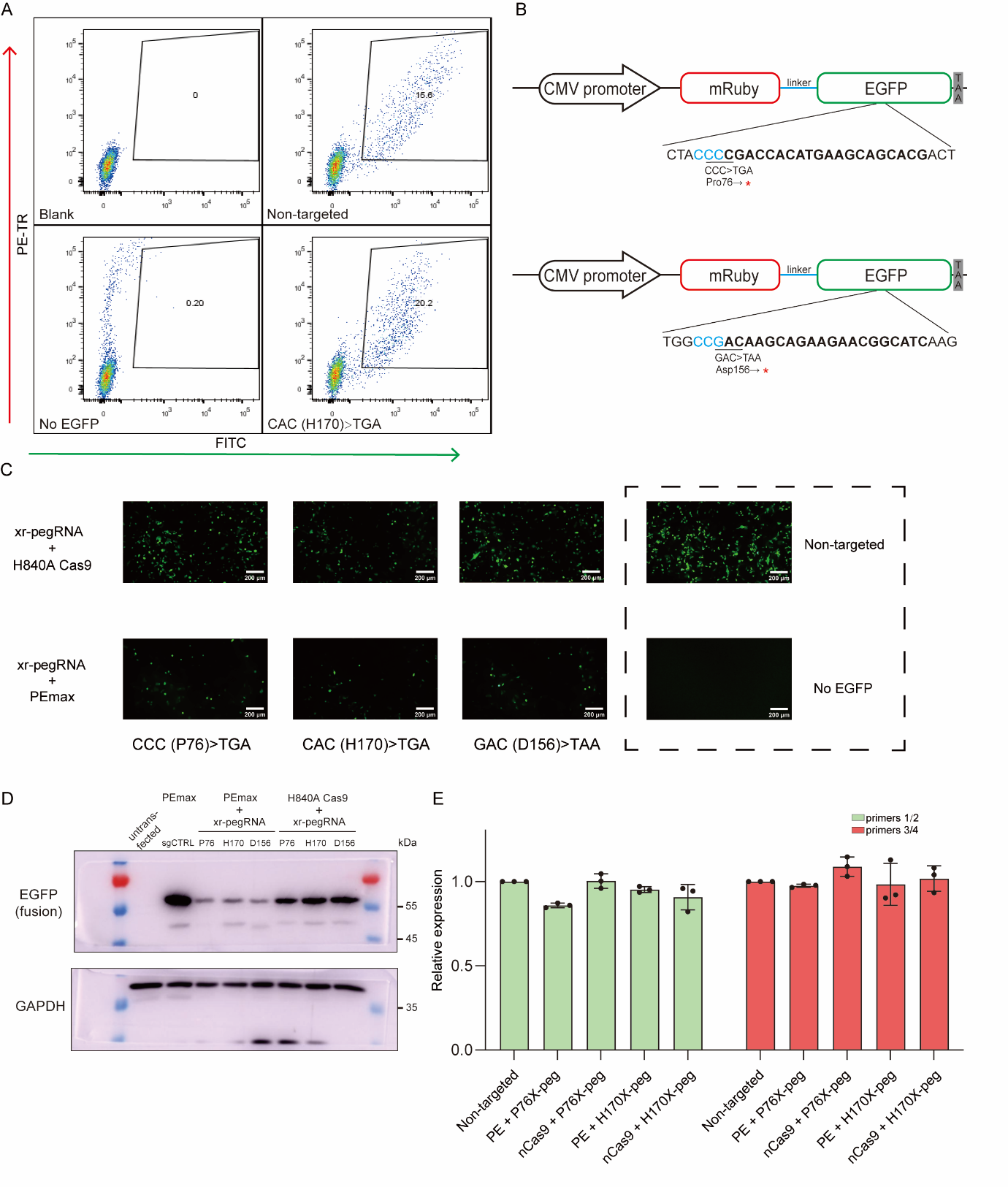
**

**Figure S4: PE-STOP could target different sites in a reporter for installation of nonsense mutations.**

1. FACS analyses of HEK293T cells introduced with PE-STOP [“CAC (H170)>TGA”] were carried out. The results from the experimental group and the control groups are shown. The non-targeted group corresponds to transfections with a random sgRNA and PEmax. Cells transfected with a plasmid with a disabled EGFP unit (mRuby-linker[TAG]-EGFP) and the untransfected cells were included as additional controls. The representative results from three biological replicates are shown in the figure.
2. A simplified scheme shows the sequence information of the PE-STOP-targeted additional codons [CCC (P76) and GAC (D156)] in the reporter. PAM sequences are marked in blue and the spacer sequences are labeled in bold. The target codons are underlined. The designed installations of stop codons are shown by red asterisks.
3. The levels of reporter fluorescence in cells introduced with PE-STOP or control plasmids are shown (fluorescent microscopy, scalebar: 200 μm). The applications of PE-STOP with different pegRNAs were accompanied by their respective control groups (transfected with the nCas9 [no RT] and the pegRNAs). Cells transfected with scrambled sgRNA, PEmax, and the reporter plasmid were used as the non-targeted control. Cells transfected with a plasmid with a disabled EGFP unit (mRuby-linker[TAG]-EGFP) were also included (no EGFP).
4. Western blot was performed with samples corresponding to cells in (C), except for the sample from “no EGFP” cells.
5. The RNA samples corresponding to PE-STOP or control targeting at CCC (P76) and CAC (H170) were analyzed by qPCR. Two prime sets (1/2 or 3/4) were employed for the analyses.

**
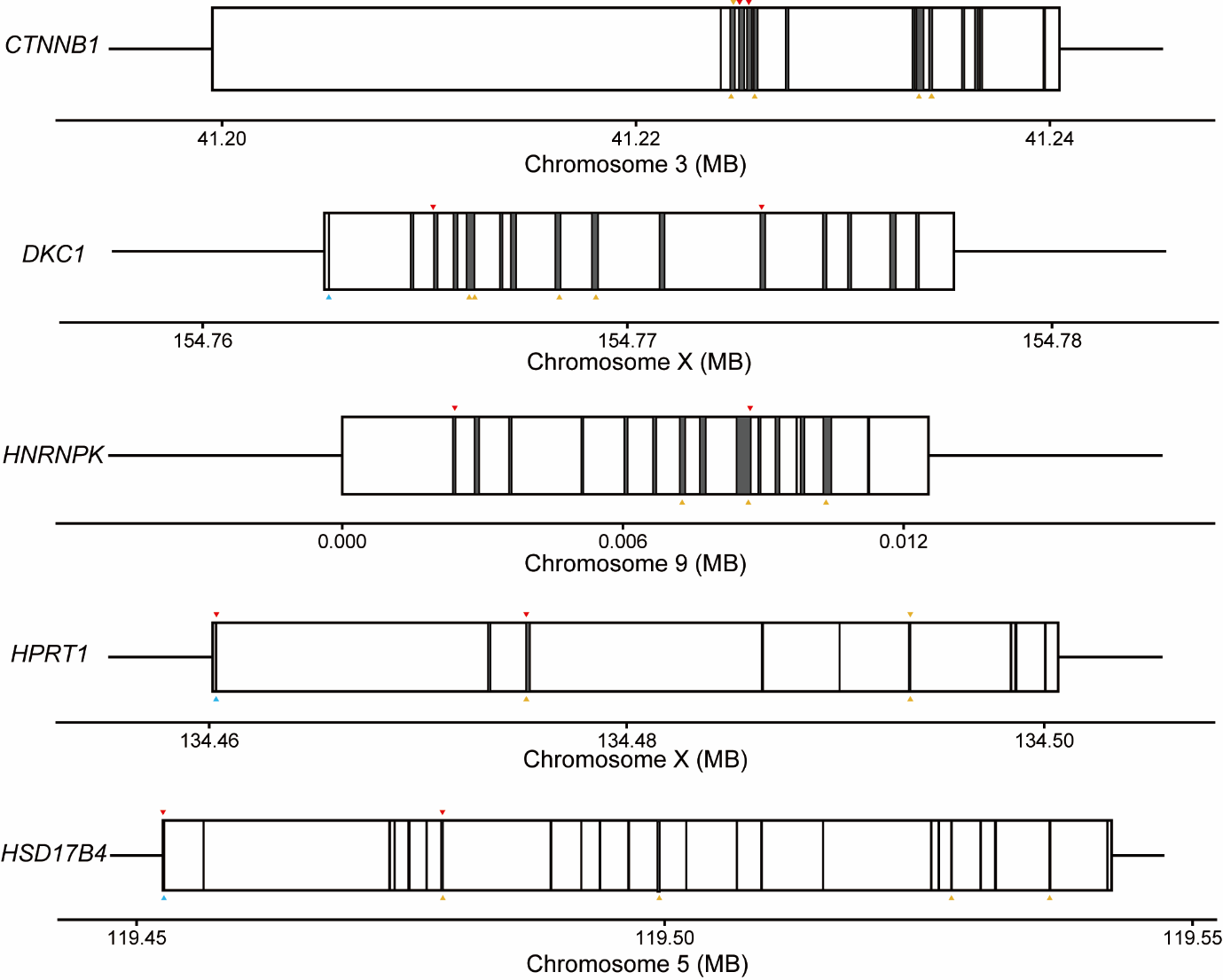
**

**Figure S5: Guide RNA designs for targeting genes in their genomic contexts into stop codons.**

The simplified schemes show the target sites for pegRNAs (PE-STOP) and sgRNAs (i-Silence and iSTOP) in five genes. The red, yellow and blue triangles mark the target sites by PE-STOP, iSTOP and i-Silence, respectively. The black boxes and white boxes represent exons and introns, respectively.

**
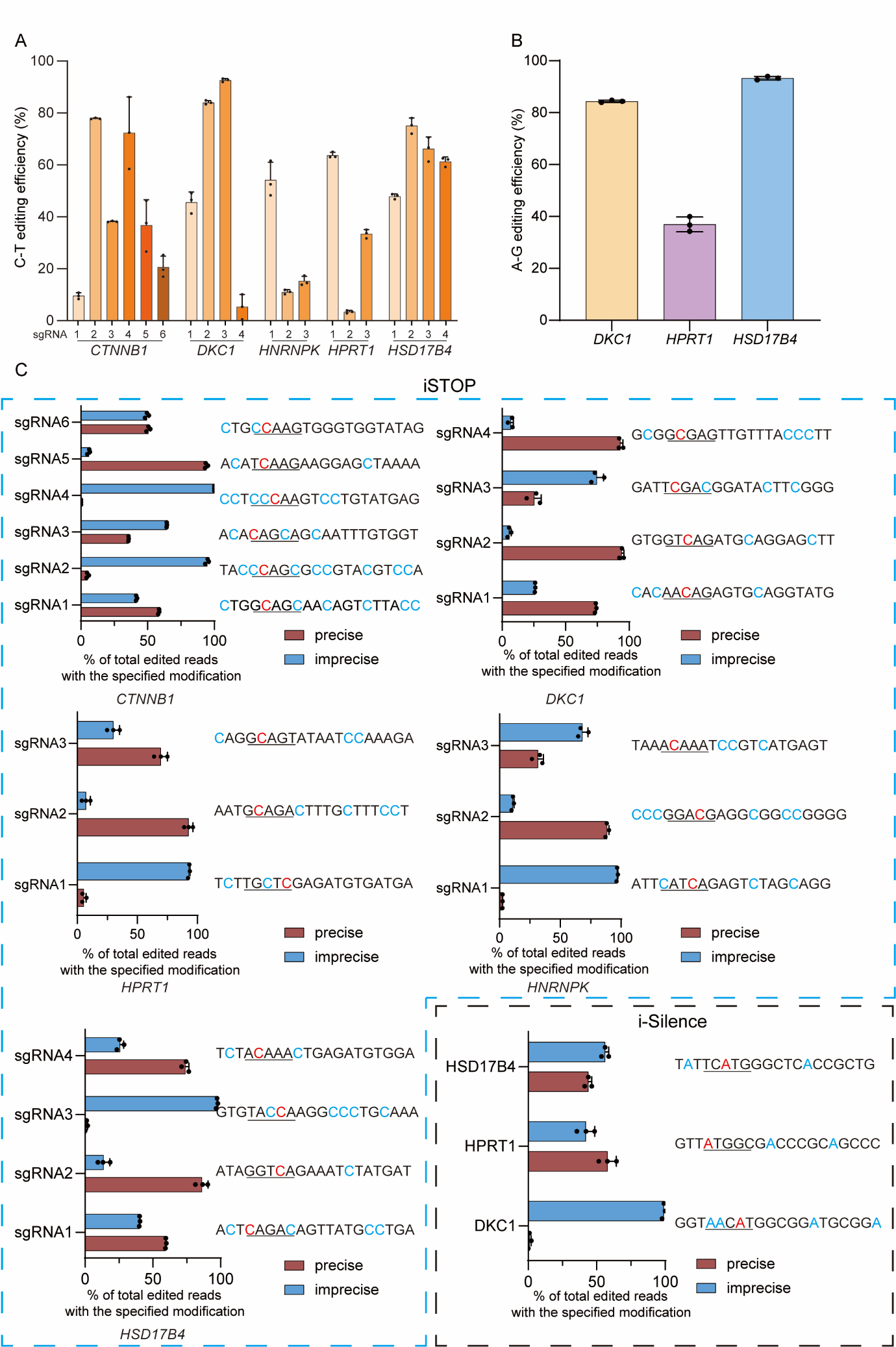
**

**Figure S6: Efficiencies and types of edits enabled by iSTOP and i-Silence at their target sites in HEK293T cells.**

1. HEK293T cells were introduced with iSTOP (with a core of AncBE4max) for targeting various sites (with CAG, CAA or CGA codons) in five genes. The overall editing efficiencies were determined by deep sequencing.
2. HEK293T cells were introduced with i-Silence (with a core of ABE8e) for targeting the start codons in three genes. The overall editing efficiencies were determined by deep sequencing. Data presented in (A and B) were derived from three biological replicates (mean ± SD).
3. The distributions of precise and imprecise edits at each target site (in both [A and B]) by the BE-dependent tools are shown. The 20-bp spacer sequences are listed near each set of data bars. The classical editing windows are underlined (set as from position 4 to 8). The target bases were labeled in red. In addition, all other potentially convertible bases within the spacer sequences are marked in blue.

**
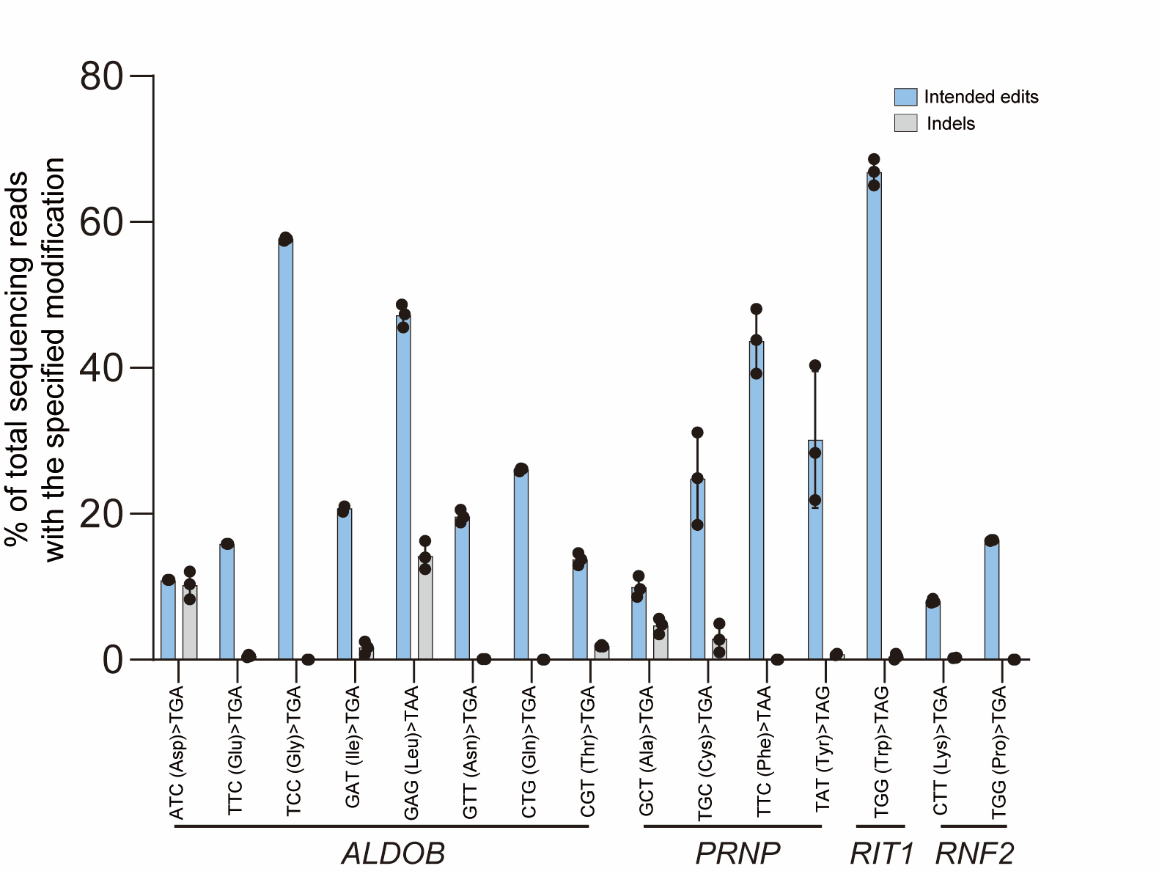
**

**Figure S7: An additional survey of pegRNA efficiencies by PE-STOP for four target genes in HEK293T cells.**

The efficiencies and the associated indel levels by PE-STOP for generation of stop codons at more target sites (each aimed at a different codon) within 4 endogenous genes. The editing efficiencies were determined by deep sequencing. The results were obtained from three biological replicates. The data are presented as mean values ± SD.

**
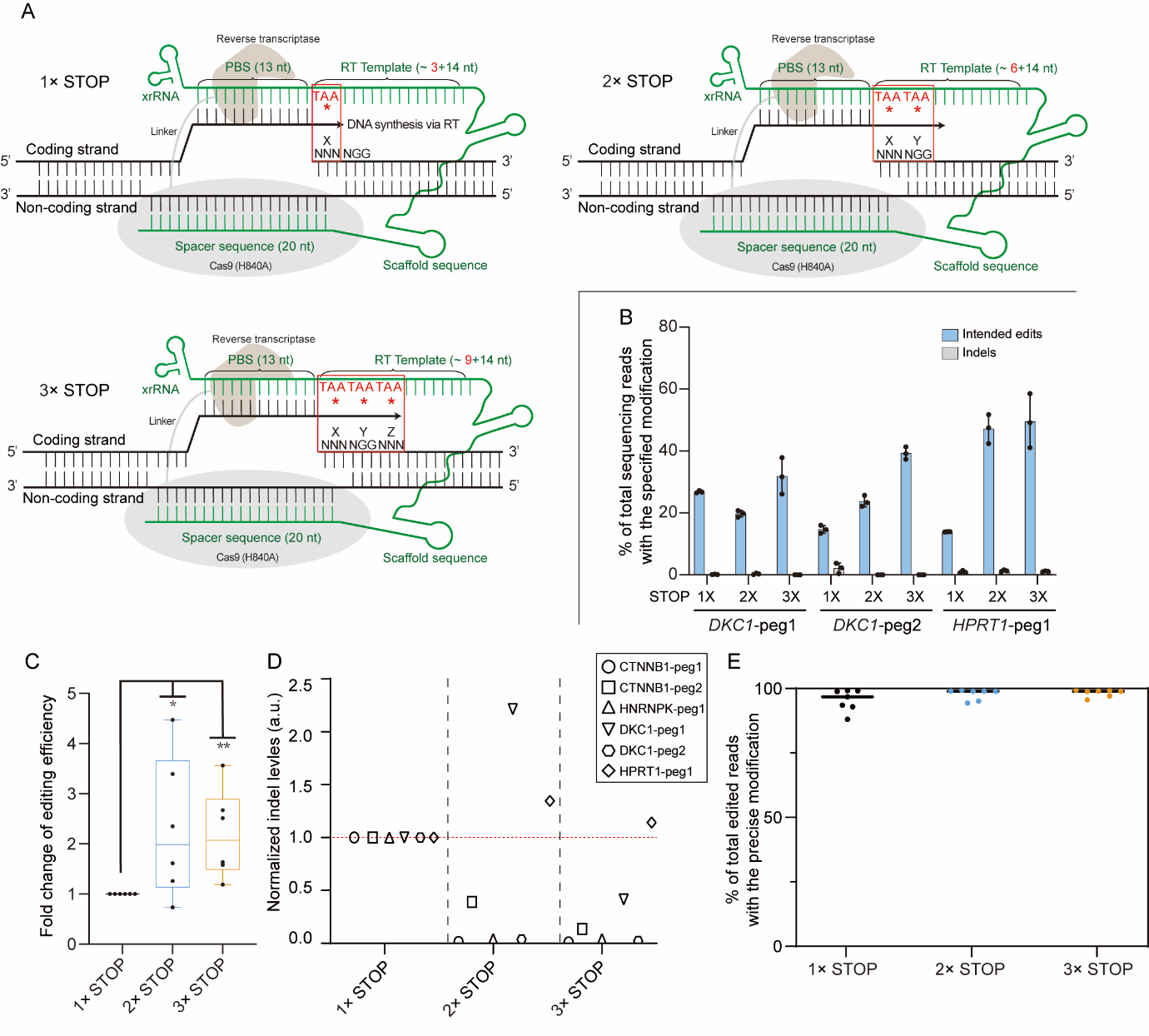
**

**Figure S8: PE-STOP can enable efficient installation of consecutive stop codons at the target sites.**

1. The illustration shows the introduction of single, double or triple stop codons with PE-STOP. The nicked target DNA is depicted, with the cleavage site (denoted as +1 position) and PAM sequence (NGG, ending at the +6 position) indicated. The pegRNA molecule is shown in green. The segments corresponding to the spacer, scaffold, RTT and PBS are respectively marked. The 1-3x “TAA”s within the RTT represent the programmed edits for installing 1-3 stop codons. For simplicity, positions respectively corresponding to +1 to +3, to +6 and to +9 in the DNA are shown as the targeted codons.
2. PE-STOP enabled installation of single, double or triple stop codons by PE-STOP at sites within *DKC1* and *HPRT1*. For installing various numbers of stop codons, the pegRNAs featured the same spacer and PBS sequences, but different length of RT sequences. Data were obtained from three biological replicates. The bar graph indicates the efficiencies and the associated indel levels under each editing conditions (mean ± SD).
3. The overall efficiencies for installation of single, double or triple stop codons by PE-STOP at six sites are summarized (see Fig. 3E and [B] above). The box plot shows the normalized editing efficiencies (over the respective efficiencies with a single stop codon). Each data point represents the averaged editing efficiency for each condition. The center line shows the medians and the box limits correspond to upper the lower quartiles, while the whiskers mark the largest and smallest points. The variances between the efficiencies for the single- and multiple (2x and 3x)-stop groups were analyzed via Student’s t-tests (*: *P*<0.05, **: *P*<0.01).
4. The indel levels in samples shown in (C, six sites) are summarized. The indel levels for each targeting (mean levels from biological triplicates) were normalized to the corresponding 1x STOP groups. The 2x and 3x STOP groups are generally associated with reduced indel levels.
5. The editing purities of the above samples (corresponding to six sites) are also summarized. The editing purities are indicated by the percentages of reads harboring only the targeted mutations (perfect edits) within all edited reads.

**
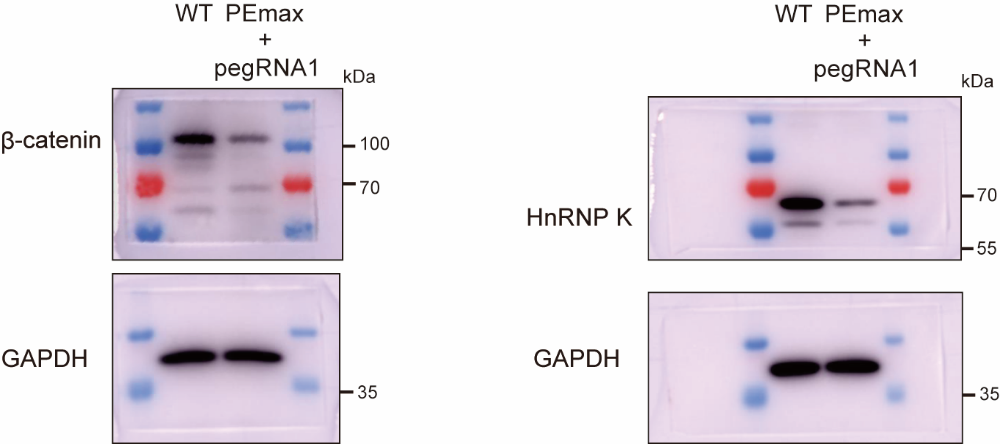
**

**Figure S9: Transient transfection of PE-STOP led to downregulation of target proteins.**

Western blot results show the levels of β-catenin and HnRNP K expression in HEK293T cells after transient transfection of PE-STOP components. The levels of GAPDH are shown as protein loading control.

**
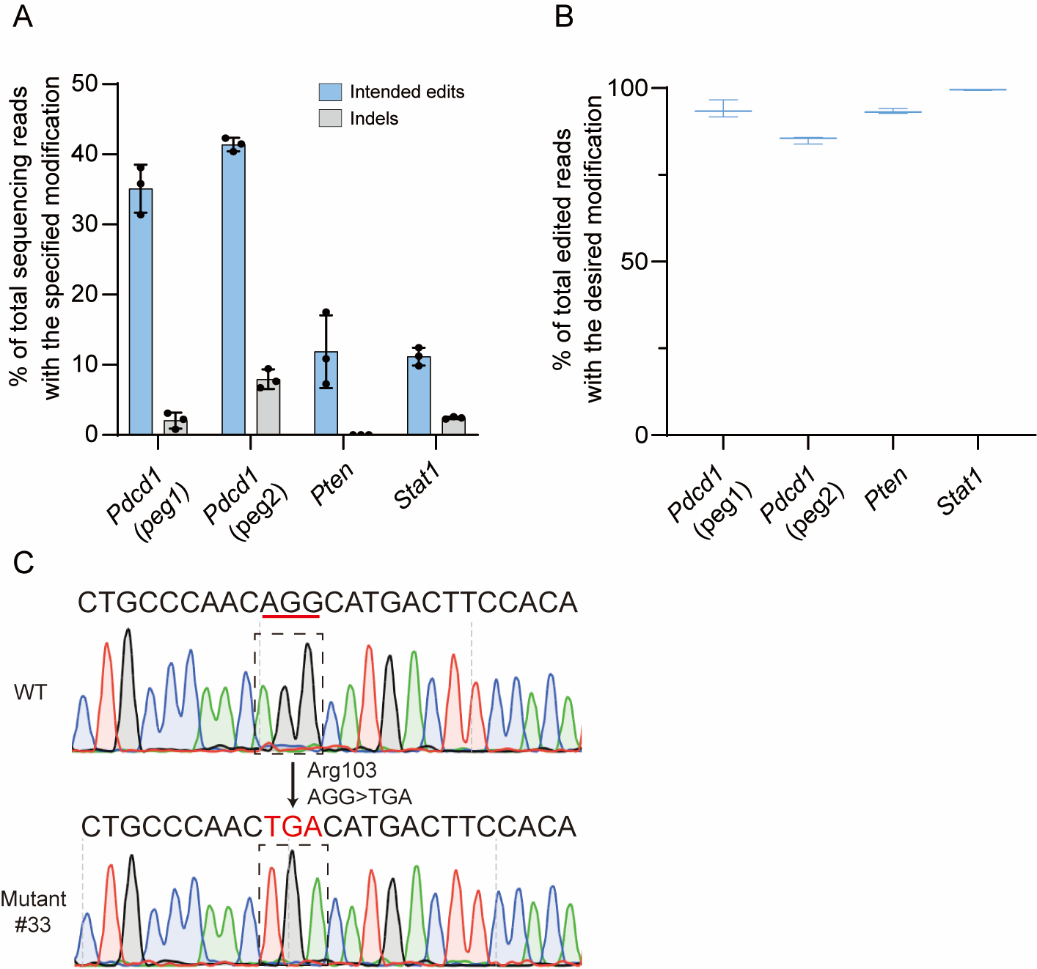
**

**Figure S10: PE-STOP enables installation of nonsense mutations in N2a cells.**

1. The editing efficiencies by PE-STOP for targeting four different sites (within three genes) are shown. The editing efficiencies and the associated indel levels were determined from the deep sequencing data (n= three biological replicates). Data are presented as mean values ± SD.
2. The levels of on-target precision by PE-STOP at different sites (see [A]). Reads harboring perfect target mutations were specified to determine the editing purities.
3. Single clones were cultivated after transfection with the PE-STOP plasmids targeting *Pdcd1* (peg1). The genotypes of the clones were determined by Sanger sequencing. The results from a homozygous mutant (#33) and from a WT clone are shown. The target codon of AGG (Arg103) is underlined, and the stop codon is labeled in red.

**
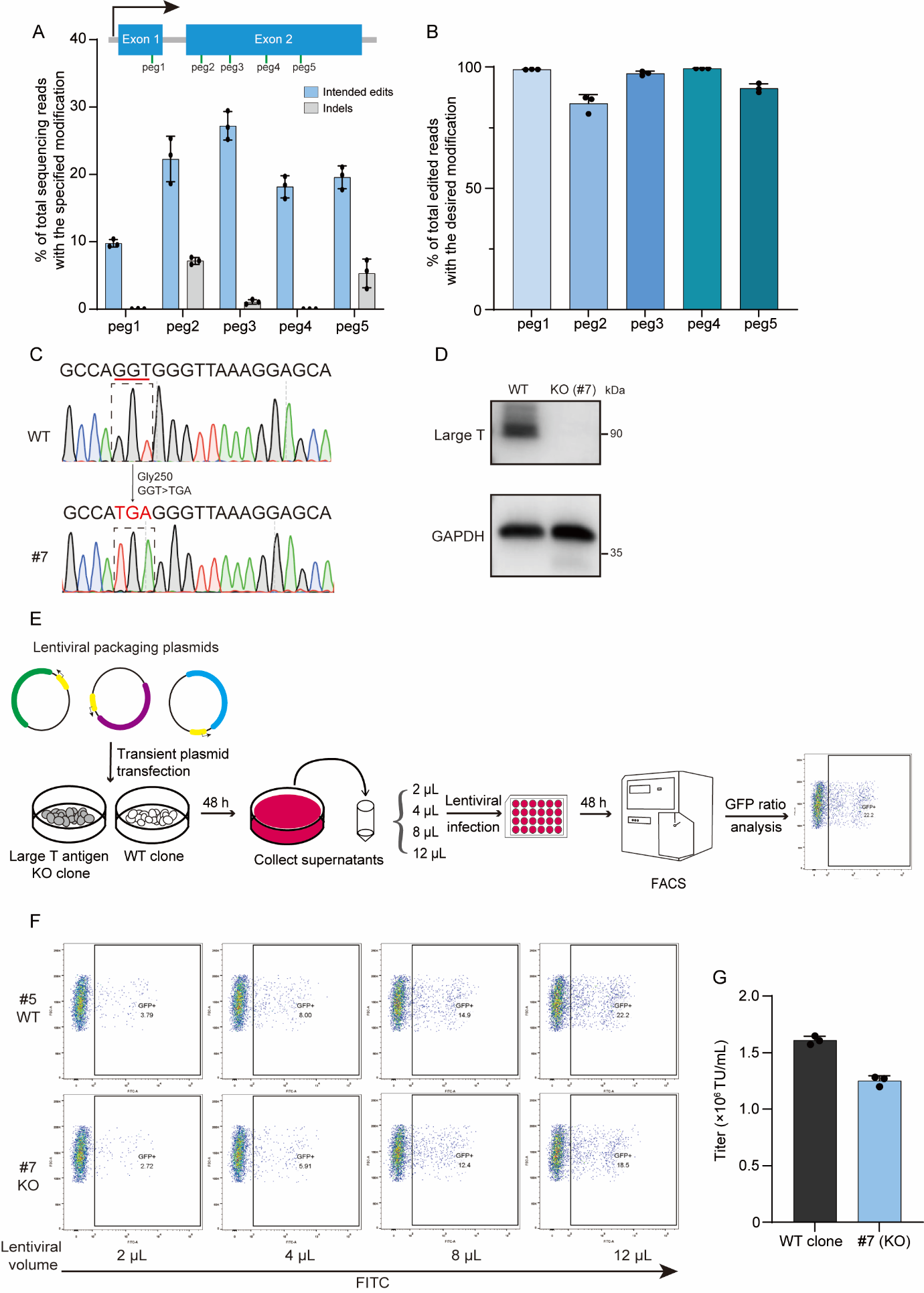
**

**Figure S11: PE-STOP-mediated inactivation of SV40 large T antigen validated its non-essentiality for efficient packaging of lentiviral vectors.**

1. The upper panel shows the targeted sites for PE-STOP at the *SV40gp6* insertion fragment (in HEK293T cells). The cells were transfected with different sets of PE-STOP plasmids. The lower panel shows deep sequencing-revealed editing efficiencies and the associated indel levels by different targeting pegRNAs.
2. The levels of on-target precision by PE-STOP at different sites. Reads harboring perfect target mutations were specified to determine the editing purities. Data presented in (A and B) were derived from measurements of three biological replicates (mean ± SD).
3. The edited cells (the “peg3” group above) were cultivated as single clones. The genotypes of the clones were determined by Sanger sequencing. The results from a homozygous mutant (#7) and from a WT clone (#5) are shown. The target GGT (Gly250) codon is underlined, and the stop codon is labeled in red.
4. The expression levels of SV40 large T antigen protein in singly cloned cells (as in [C]) were examined by Western blot as indicated. GAPDH was used as a protein loading control.
5. General workflow for estimations of lentiviral vector titers produced by different packaging cells. The transfer vector contained an EGFP expression cassette, allowing assessment of vector titer by FACS analyses.
6. Different packaging cells (single clone WT and large T antigen-inactivated cells) were used to produce lentiviral vectors. The supernatants were harvested. The indicated amounts of supernatants from the two packaging cells were used to transduce the recipient cells (parental HEK293T). FACS analyses were employed to measure the percentages of EGFP^+^ cells. The figure shows representative results from three biological replicates of transduced cells.
7. Effects of large T antigen protein inactivation on lentiviral vector productivity by HEK293T cells. After linear fitting of EGFP^+^ cell numbers to the volume of supernatants, the vector titers were determined (transduction units [TU]/mL).

**
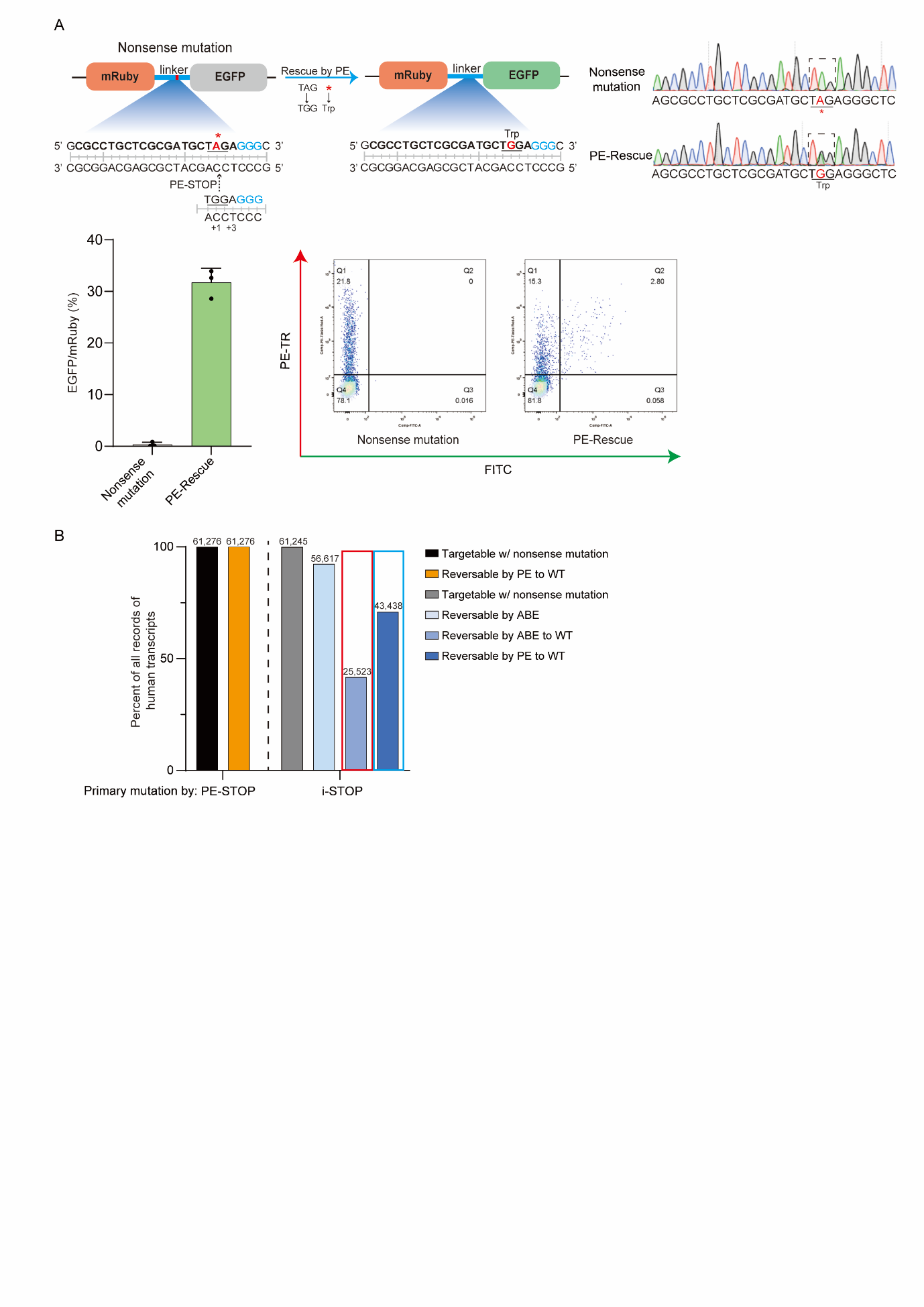
**

**Figure S12: Nonsense mutations by PE-STOP are potentially more amenable to a subsequent round of reverse mutations by PE.**

1. The illustration on top shows a scenario in which a prospective stop codon (with TAG underlined) installed by PE-STOP may be conveniently rescued by PE back to the original codon (with TGG underlined). The listed example is to induce reverse mutation of such a stop codon located in a reporter system to subsequently allow the expression of an EGFP unit. This was tested in experiments. The Sanger sequencing results (top right) indicate partial “rescue” of the stop codon into TGG. The transfected cells were analyzed by FACS. The quantitation of EGFP levels and the original dot plots are shown in the bottom panel.
2. The coverage analyses by different editors (see Figure 1B and Figure S2) were extended to the scenarios of subsequent reverse mutations. Given a total number of 61,276 human transcripts, a hit would be recorded when the sequence correspond to a transcript could be programmed for at least one series of nonsense mutation followed by a subsequent reverse mutation. For an iSTOP-induced nonsense mutations (C-to-T), the possibility of reverse mutations by ABE (A-to-G) on the opposite strand is presented with the light-blue bar. As ABE may trigger bystander editing, the possibility of reverse mutations by ABE to restore the WT sequences is presented with the medium-blue bar. Furthermore, the possibility of reverse mutation by PE to restore the WT sequence is presented with the dark-blue bar.

**
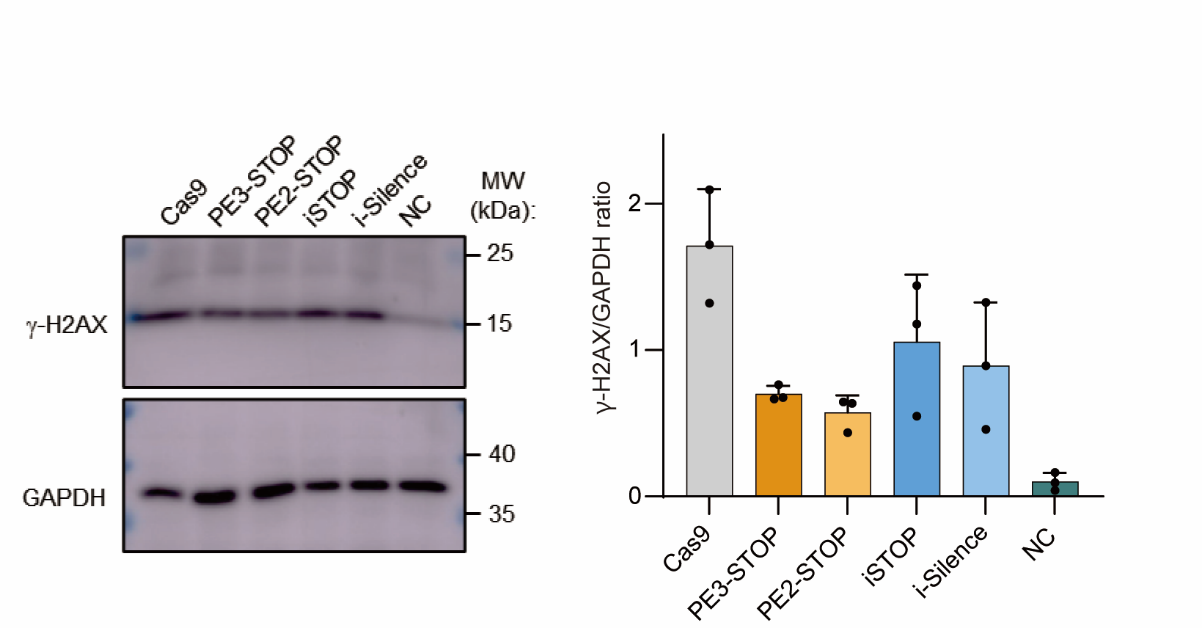
**

**Figure S13: The application of PE-STOP was associated with lower induction of γ-H2AX than that by iSTOP or i-Silence.**

HEK293T cells were transfected with the plasmids corresponding to editing by Cas9, iSTOP, i-Silence and PE-STOP (PE3 and PE2). Except for i-Silence that specifically targeted the start codon, all other editing platforms targeted the same site in *HPRT1* gene (corresponding to peg2 and sgRNA1). A representative immunoblot of γ-H2AX and GAPDH (loading control) in the cell lysates is presented on the left. Quantitation of results from three independent experiments is shown on the right.
